# Optimization of Molecular Approaches to Genogroup *Neisseria meningitidis* Carriage Isolates and Implications for Monitoring the Impact of New Serogroup B Vaccines

**DOI:** 10.1371/journal.pone.0132140

**Published:** 2015-07-06

**Authors:** Eduardo Rojas, Johanna Hoyos, Neil J. Oldfield, Philip Lee, Mike Flint, C. Hal Jones, Dlawer A. A. Ala’Aldeen, Kathrin U. Jansen, Annaliesa S. Anderson

**Affiliations:** 1 Vaccine Research and Development, Pfizer Inc, Pearl River, New York, United States of America; 2 School of Life Sciences, University of Nottingham, Nottingham, United Kingdom; Melbourne School of Population Health, AUSTRALIA

## Abstract

The reservoir for *Neisseria meningitidis* (Nm) is the human oropharynx. Implementation of Nm serogroup C (NmC) glycoconjugate vaccines directly reduced NmC carriage. Prophylactic vaccines are now available to prevent disease caused by the five major Nm disease causing serogroups (ABCWY). Nm serogroup B (NmB) vaccines are composed of antigens that are conserved across Nm serogroups and therefore have the potential to impact all Nm carriage. To assess the effect of these vaccines on carriage, standardized approaches to identify and group Nm are required. Real-time PCR (rt-PCR) capsule grouping assays that were internally controlled to confirm Nm species were developed for eight serogroups associated with carriage (A, B, C, E, W, X, Y and Z). The grouping scheme was validated using diverse bacterial species associated with carriage and then used to evaluate a collection of diverse Nm carriage isolates (*n*=234). A scheme that also included *porA* and *ctrA* probes was able to speciate the isolates, while *ctrA* also provided insights on the integrity of the polysaccharide loci. Isolates were typed for the Nm vaccine antigen factor H binding protein (fHbp), and were found to represent the known diversity of this antigen. The *porA* rt-PCR yielded positive results with all 234 of the Nm carriage isolates. Genogrouping assays classified 76.5% (179/234) of these isolates to a group, categorized 53 as nongenogroupable (NGG) and two as mixed results. Thirty seven NGG isolates evidenced a disrupted capsular polysaccharide operon judged by a *ctrA* negative result. Only 28.6% (67/234) of the isolates were serogrouped by slide agglutination (SASG), highlighting the reduced capability of carriage strains to express capsular polysaccharide. These rt-PCR assays provide a comprehensive means to identify and genogroup *N*. *meningitidis* in carriage studies used to guide vaccination strategies and to assess the impact of novel fHbp containing vaccines on meningococcal carriage.

## Introduction

Meningococcal infection is a major cause of bacterial meningitis and septicemia worldwide. *Neisseria meningitidis* (Nm) commonly colonizes the oropharyngeal mucosa without causing any detectable symptoms [1]. Most individuals will, at one time or another during their lives, carry the bacterium asymptomatically in their throats (a condition defined as carriage). Invasive disease is rare in non-epidemic areas such as Europe and the US, occurring at a rate of 0.5–10 cases per 100,000 population per year [2]. However, before the implementation of serogroup A glycoconjugate vaccines, rates of up to 1,000 cases per 100,000 population per year had been recorded in the epidemic region of sub-Saharan Africa [3–6].

Nm produces a polysaccharide capsule that is exhibited on the cell surface of invasive isolates and helps protect the bacterium from the human immune system [7]. Nm can be classified into serogroups based on the structure of its polysaccharide capsule and the use of polysaccharide-specific antisera. Twelve serogroups have been described (A, B, C, E, H, I, K, L, W, X, Y and Z), but only five (A, B, C, W and Y) are responsible for greater than 90% of the invasive disease worldwide. Several prophylactic capsular polysaccharide and polysaccharide-conjugate based vaccines against serogroups A, C, Y, and W have been developed and licensed [8], however similar approaches to develop a *N*. *meningitidis* serogroup B (NmB) vaccine have failed. The serogroup B capsular polysaccharide is composed of polysialic acid repeating units that are similar to structures found on human neuronal cells (particularly during fetal development) and are not suitable as vaccine antigens [9]. One alternate strategy to prevent NmB disease focused on vaccines produced from bacterial outer membrane vesicles (OMVs). However, due to considerable sequence heterogeneity of the immunodominant PorA vesicle antigen, OMV vaccines are not suitable in endemic situations where broader protective efficacy is required [10]. Several non-capsular NmB proteins have been considered as antigens for a broadly effective vaccine and the surface lipoprotein factor H binding protein (fHbp) has emerged as a leading candidate. Molecular epidemiology of NmB clinical isolates demonstrated that *fhbp* gene sequences segregate into two subfamilies, designated A and B [11]. Protein variants within subfamilies share >83% amino acid identity, but only 60%–75% pairwise identity between subfamilies [12]. Consistent with the sequence diversity between subfamily A and B, the breadth of bactericidal activity following immunization with lipidated fHbp antigens is subfamily restricted [11,13]. Two vaccines, each containing fHbp, have been licensed in the United States for the prevention of invasive meningococcal disease caused by NmB. The first, bivalent rLP2086 (Trumenba), is composed of two lipidated fHbp variants (A05, B01), one each from subfamily A and B to confer breadth of immune coverage. 4CMenB (Bexsero), a four component NmB vaccine that includes a non-lipidated fHbp variant from subfamily B (B24) together with two additional meningococcal proteins and OMV, has more recently been licensed in the US.

Eight meningococcal serogroups (A, B, C, E, W, X, Y and Z) are commonly detected in carriage isolates [14]. The epidemiology of meningococcal carriage and disease is the subject of ongoing studies, particularly as prophylactic vaccines have now been developed for each of the five serogroups associated with disease [15]. Implementation of the *N*. *meningitidis* serogroup C (NmC) glycoconjugate vaccine in the UK reduced not only the disease caused by this serogroup, but also its carriage rate [16] resulting in herd immunity being extended to non-vaccinated age groups [17]. The introduction of meningococcal vaccines against NmB that are based on protein antigens, such as factor H binding protein (fHbp), may also impact other Nm serogroups [18,19]. The development of standardized rapid and robust approaches to assess the impact of these vaccines on meningococcal carriage is desirable. Preliminary studies conducted with Bexsero, have demonstrated that although the vaccine failed its primary endpoint for the reduction of carriage, it may have an effect on carriage strain acquisition over time. Overall, an 18% reduction in carriage was reported for all isolates [15]. In this study, carriage isolates were grouped using a combination of serogrouping and genogrouping approaches to characterize the disease associated serogroups (ABCWY). In contrast to invasive strains, many meningococcal isolates obtained from asymptomatic carriers lack capsule expression and are therefore ungroupable by serology [20,21]. For that reason, a comprehensive strategy aimed at characterizing carriage isolates must include molecular in addition to serological methods. Polymerase chain reaction (PCR) is a fast and sensitive method that has been used both in clinical diagnostics and in the management of infectious diseases since the mid-1990s. It is the preferred non-culture method for identifying the causal agent of meningitis in the clinic, especially since the early administration of antibiotics often prevents the recovery of viable bacteria [22,23]. In contrast to conventional gel-based PCR assays, real-time PCR (rt-PCR) provides higher sensitivity, speed, throughput and prospective automation, all desirable features for epidemiological studies. In addition, closed-tube analysis eliminates the risk of potential contamination during post-PCR handling steps.

Reliable detection methods should also include assay controls that confirm the presence of Nm in the assay. Assays for a variety of Nm specific loci have been described in the literature. These include insertion elements, 16S ribosomal RNA, the capsule regulatory gene *crgA*, the capsule transport gene *ctrA*, *porA*, and *sodC* [22,24–29]. The gene encoding CtrA is the most widely used target for meningococcal identification in invasive disease. However, there are few studies to demonstrate that this is an appropriate approach for carriage isolates which may have incomplete or absent capsular polysaccharide operons [30]. Jordens and Heckels reported the development of a *porA* rt-PCR assay that identified several isolates that were missed by using only *ctrA* [28]. To increase accuracy and prevent false negative results, Whiley and collaborators used a duplex rt-PCR approach that targeted both *porA* and *ctrA* [31]. Although this is successful at detecting most Nm invasive strains, some may still be missed due to sequence polymorphisms [32,33]. The reported *sodC* assay [29] was effective at identifying 99.7% (624/626) of invasive and carriage Nm strains, and was superior to the *ctrA* assay (71.6%, 448/626). The 28.4% (178/626) of isolates that were not identified as Nm using the *ctrA* assay was comprised entirely of nongroupable (NG) strains.

**Fig 1:**
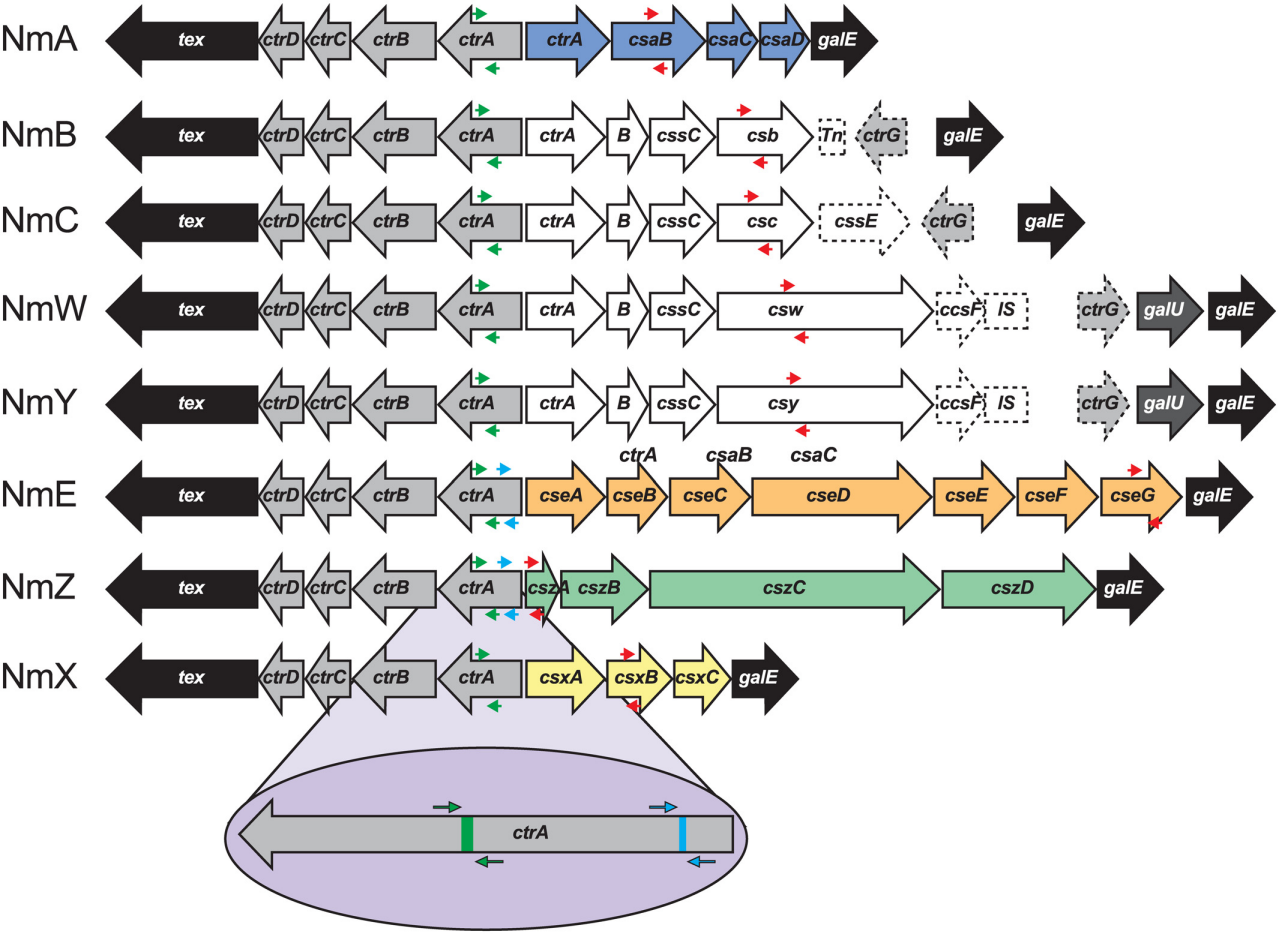
Location of serogroup-specific PCR amplicons in capsular biosynthesis genes of *N*. *meningitidis* serogroups A, B, C, W, Y, E, Z, and X. Genes *ctrA-D* encode capsule transport proteins conserved among all eight serogroups. Genes *cssA-C* are responsible for the sialic acid capsular component of serogroups B, C, W, and Y, while *csb-c-w-y* encode the sialyltransferases that produce the serogroup-specific linkages of sialic acid with glucose or galactose. Capsule biosynthesis genes *csaA-D*, *csxA-C*, *cseA-G*, and *cszA-D*, are unique to A, X, E, and Z serogroups, respectively. Polysaccharide transport and biosynthesis genes are flanked by *tex* and *galE* (black arrows). Genes at the distal end of the biosynthetic clusters in serogroups B, C, W, and Y are shown as dotted arrows, and annotated as described previously [35]. The green arrows indicate the location of the *ctrA* primers used to detect all eight serogroups. Light blue arrows represent the initial primers designed to identify NmE and NmZ, based on sequence at the 5’ end of the *ctrA* gene. The red arrows represent the position of the final primers designed for the eight serogroup-specific assays. The blow-up at the bottom illustrates the position of *ctrA* primers and probes in greater detail.

PCR grouping of meningococci relies on the accurate detection of the gene(s) responsible for determining the capsular type. The enzymes that synthesize and translocate capsular polysaccharides are encoded by an operon that is composed of between seven and eleven genes flanked by *tex* and *galE* (Fig 1) [34,35]. Thus, rt-PCR assays for genotypic grouping of Nm based on the detection of serogroup-specific genes *csaB* (NmA), *csb* (NmB), *csc* (NmC), *csw* (NmW), *csxB* (NmX) and *csy* (NmY) have been described [36,37]. However, no rt-PCR assays for genogrouping NmE and NmZ have been reported [29,37,38]. We therefore sought to develop assays to identify and genogroup carriage isolates, while trying to address the shortcomings present in current assay designs. We developed optimized *porA* and *ctrA* rt-PCR assays that identified Nm and assays that grouped isolates from the eight major serogroups (A, B, C, E, W, X, Y and Z) to assist in surveying the current state of meningococcal carriage. Carriage isolates were also typed for fHbp to evaluate its distribution across the Nm carriage isolates.

## Methods

### Bacterial Strains

Three panels of bacterial isolates were employed to develop and evaluate the assays described herein. The first panel consisted of NmE (*n* = 69) and NmZ (*n* = 13) strains and was used to obtain the DNA sequences of capsule biosynthesis and transport genes. The second panel was used for proficiency to confirm the specificity of the PCR assays and was composed of: NmA (*n* = 4), NmB (*n* = 5), NmC (*n* = 3), NmE (*n* = 69), NmW (*n* = 3), NmX (*n* = 3), NmY (*n* = 5), NmZ (*n* = 9), Nm *cnl* (*n* = 6), and 38 additional strains that included other human pathogens and commensal organisms, specifically: *N*. *cinerea* (*n* = 1), *N*. *gonorrhoeae* (*n* = 2), *N*. *lactamica* (*n* = 2), *N*. *sicca* (*n* = 2) and *N*. *subflava* (*n* = 2); *Haemophilus influenzae* [type *a* (*n* = 1), *b* (*n* = 2), *c* (*n* = 2), *d* (*n* = 1), *e* (*n* = 1), *f* (*n* = 1), *i* (*n* = 1), *non-typeable* (*n* = 2), biogroup *aegyptius* (*n* = 1)], *H*. *haemolyticus* (*n* = 1) and *H*. *parainfluenzae* (*n* = 2); *Streptococcus pyogenes* (*n* = 1), *S*. *agalactiae* (*n* = 2), *S*. *pneumoniae* type 1, 5, 14 and 19F (one of each; *n* = 4); *Staphylococcus aureus* (*n* = 2) and *Moraxella catarrhalis* (*n* = 5). These strains were obtained either from ATCC (Bethesda, MA) or from Pfizer’s collection of clinical isolates.

The third panel was composed of *Neisseria* carriage isolates (*n* = 234) that were selected in a non-biased manner from strains collected over the course of two studies that investigated meningococcal carriage in the UK during 1997–2001 (*n* = 2296). The first study was conducted at the University of Nottingham [39] and the second was carried out in a multicenter survey of carriage during the introduction of a NmC conjugate vaccine and on two successive years [16]. These isolates were previously grouped [16,39] using a standard PCR assay.

*Neisseria* strains were grown overnight on chocolate agar plates at 37°C under 5% CO_2_ and cell lysates were generated by heating a loopful of colonies suspended in 100 μL H_2_O for 5 min at 95°C. Lysates were centrifuged at 14,000 × *g* for 5 min and an aliquot was diluted 20- to 50-fold in TE buffer pH 8.0 (10 mM Tris-HCl pH 8.0, 1 mM EDTA) for testing.

### Genomic DNA Extraction

Bacteria from an overnight chocolate agar culture were collected in 3 mL sterile PBS. A 500 μL aliquot of this suspension was lysed and processed using the DNeasy Blood and Tissue kit (Qiagen, Valencia, CA) as recommended by the manufacturer. DNA was eluted in 200 μL of Buffer EB and its concentration determined by measuring A_260_ using a Nanodrop ND-1000 spectrophotometer (Nanodrop Tech, Wilmington, DE). DNA integrity was evaluated by running 500 ng in a 1% agarose gel. DNA standards were prepared by making six 10-fold serial dilutions ranging from 20 to 2×10^6^ genome copies per microliter and were used to determine efficiency and the lower limit of quantitation for each rt-PCR assay.

### Alignments and Primer-Probe Design

Real-time PCR primers and probes were designed using DNASTAR Lasergene 10 and Primer Express V 3.0. (Life Technologies, Grand Island, NY) and are listed in Table 1. Conserved and discriminating regions within target genes were identified based on nucleotide sequence alignments of *N*. *meningitidis* sequences retrieved from GenBank or from Pfizer’s collection of clinical isolates. For meningococcal identification, primer-probe sets were targeted to conserved regions of genes encoding 16S rRNA, *crgA*, *porA* and *ctrA*. We designed primer-probe sets for each of these four genes and evaluated them with serial dilutions of NmC and NmY genomic DNA to calculate PCR efficiency and sensitivity. Genogrouping PCR primers and probes developed by other investigators [36] were first evaluated *in silico*. Then, alignments of GenBank sequences for *csaB* (NmA), *csb* (NmB), *csc* (NmC), *csw* (NmW), *csy* (NmY), and *c*s*xB* (NmX) were used to design Taqman Minor Groove Binder (MGB) probes for genogrouping *N*. *meningitidis* A, B, C, W, X, Y and Taqman probes for serogroups E and Z (Table 1). Initial NmE and NmZ primers and probes were selected to target the 5’end of the *ctrA* gene, which harbours nucleotide sequences capable of discriminating serogroups E, X, and Z [40]. Alignments of NmE and NmZ *ctrA* sequences extracted from GenBank (AF520907 and AF520909, respectively) together with *ctrA* sequences generated in house from 64 NmE and 12 NmZ isolates, guided the design of group E and Z specific primers and probes. Later, an improved set of primers and probes targeting capsule biosynthesis genes *cszA* (NmZ) and *cseG* (NmE) were designed based on alignments of *cszA* sequences from eight NmZ isolates (described in the next section) to Genbank sequence AJ744766 (NmZ) and *cseG* sequences from 64 NmE isolates to Genbank sequence AJ576117 (NmE), respectively.

**Table 1:** Real-Time PCR Primers and Probes for Identification and Genogrouping *N*. *meningitidis*.

Serogroup	Gene	Name	Sequence 5’→3’	Ct LOD^b^
**All**		Nm16S-F913	CGGTGGATGATGTGGATTAATTC	
	16S rRNA	Nm16S-R986	CCGGAGGATTCCGTACATGT	34.40
		Nm16S-P947	FAM-AAGAACCTTACCTGGTCTT-MGBNFQ	
**All**		NmcrgA-F484	AGCCGCTTCCGCGTAATC	
	*crgA*	NmcrgA-R565	GGTGACCGGCAAGCTCTTC	32.8
		NmcrgA-P505	FAM-AGTCCTGAATACCTGGC-MGBNFQ	
**All**		NmporA-F928	CGCTTCGGTAATGCAGTTCC	
	*porA*	NmporA-R1055	CGTTTGGAAAAATCATAATCAACG	35.28
		NmporA-Pr1009	FAM-TGGTATTTTCGCCTTTTTTACCGCGTT-BHQ-1	
**All**		NmctrA-F860	TGGGCGGTTTGCAAGATC	
	*ctrA*	NmctrA-R947	TGACGTTCTGCCGGCAAT	33.81
		NmctrA-Pr900	VIC-CACACCACGCGCATCA-MGBNFQ	
**A**		NmAsacB-F1150	GCCACAAAGTGCCCTTCCT	
	*csaB*	NmAsacB-R1213	TGGTATATGGTGCAAGCTGGTT	35.48
		NmAsacB-P1172	NED-TTTAGCTCACATGCTATTG-MGBNFQ	
**B**		NmBsiaD-F895	CCTCGGCTGGTAGTTATTAATGAAC	
	*csb*	NmBsiaD-R980	GCCAGGCCTATAATTCCTTTAGGA	33.35
		NmBsiaD-P922	FAM-CCTTTTCTAATTGAGCCCCTAA-MGBNFQ	
**C**		NmCsiaD-F496	GCACATTCAGGCGGGATTA	
	*csc*	NmCsiaD-R564	TTGAGATATGCGGTATTTGTCTTGA	34.84
		NmCsiaD-P517	NED-ACAAGCCAATCTATTGCT-MGBNFQ	
**X**		NmXxcbB-F364	TTGCGCCGCCATAAAGA	
	*csxB*	NmXxcbB-R428	GAGGCAGATTTGCGTTTTGG	n.a
		NmXxcbB-P393	FAM-ACTGACACCACCTTC-MGBNFQ	
**W**		NmWsynG-F872	TGGTGTATGATATTCCAATCGTTGT	
	*csw*	NmWsynG-R982	CCATTCCAGAAATATCACCAGTTTT	34.00
		NmWsynG-P915	VIC-AGCGAATGATTACAGTAACT-MGBNFQ	
**Y**		NmYsynF-F954	GTACGATATCCCTATCCTTGCCTATAA	
	*csy*	NmYsynF-R1060	CCATTCCAGAAATATCACCAGTTTTA	34.66
		NmYsynF-P990	VIC-TGGAGCGAATGATTTTAGCAA-MGBNFQ	
**E**		NmE ctrA-F113	ACGGCATGTGGAGCCATT	
	*ctrA*	NmE ctrA-R175	TTGCCCTAAGGAGACAATCTTTTT	33.17
		NmE ctrA-P132	FAM-CTTCCTCAGGTCCAAGTG-MGBNFQ	
**E** ^a^		capEH-F738	AGATTTACGTCGTGCCTTCGA	
	*cseG*	capEH-R865	CATGCATATAGGCTTCAGCTTCTG	30.75
		capEH-P810	FAM-TCCTAAATATGTTTCTGCTGATGCCCGC-NFQ	
**Z**		NmZ ctrA-F60	TGGTTACGGCATGTAGTGCTATTC	
	*ctrA*	NmZ ctrA-R129	TGTTGCCCTAAAGAGACAATTTTTT	33.49
		NmZ ctrA-P85	FAM-CTCCTCCGGCCCTAG-MGBNFQ	
**Z** ^a^		capZA-F39	GCACTATGGTCATATTCGCCTTT	
	*cszA*	capZA-R116	TCATCAGTTGAGATGGCAACAGT	30.84
		capZA-P65	FAM-AACGTGCAAAAGCTCTAGGCGACCATC-NFQ	

MGB = minor groove binder; NFQ = non-fluorescent quencher; BHQ-1 = Black Hole Quencher 1. Probes were fluorescently labeled with FAM, VIC or NED.

^a^ Higher specificity; located in group-specific capsule biosynthesis gene.

^b^ Ct resulting when 100 genome copies tested (lowest standard), except for 16S, which is the result of 400 genome copies because *Nm* carries four copies of 16S per genome.

To confirm specificity, all primer and probe sequences were used as queries in BLAST searches against the GenBank nucleotide database. If one primer or probe displayed high homology to other targets or species, the specificity relied on the second primer and probe in the assay to diminish the risk of generating nonspecific results. Primers were manufactured by Integrated DNA Technologies, Inc. (Coralville, IA). Probes labeled with FAM, VIC or NED and non-fluorescent quenchers (NFQ) were manufactured by Life Technologies. Real-time PCR assays were optimized using both genomic DNA and plasmids containing the target region as templates. The testing of genomic DNA from meningococcal strains of serogroup A, B, C, E, W, X, Y and Z determined that the assays were serogroup specific. All assays met the acceptable PCR efficiency criteria of 90–110%. Their efficiency ranged from 92.3% to 103.1% as calculated from standard curves resulting from testing 10-fold serial dilutions of template (1x10^7^ to 100 genome copies). Using data from the same standard curves we determined the assay sensitivity to be at least 100 genome copies.

### Cloning and Sequencing of the *cps* Biosynthesis Locus of NmZ and *cseG* from NmE

Eight isolates from our collection (PMB397, PMB667, PMB1473, PMB1867, PMB358, PMB828, PMB562 and PMB3083) identified as NmZ based on the sequence of their *ctrA* gene, were chosen for PCR amplification of region A (~8.5 kb) of the capsule biosynthesis locus (Fig 1). Five microliters of lysate were amplified for 30 PCR cycles with 200 nM of each primer ctrAUR2 (5’-TTTGTCGCGGATTTGCAACTA-3’) and NmZgalE rev (5’-CCGGTGCCGCCGGTAA-3’), 25 μL 2x TaKaRa ExTaq PCR master mix (Clontech Laboratories, Inc., Mountain View, CA) in a 50-μL reaction. Each cycle included 10 s at 98°C, 30 s at 60°C and an 8 min extension step at 68°C. PCR products were gel purified using the MinElute Gel Extraction Kit (Qiagen) and 5–50 ng was ligated into pSC-A using the StrataClone PCR cloning kit (Stratagene, La Jolla, CA). The resulting plasmids were sequenced by Sanger chemistry in an ABI 3730 (Life Technologies) using a series of forward and reverse primers based on the GenBank reference sequence AJ744766. Contigs were assembled and analyzed using Sequencher software 4.10.1 (Gene Codes, Ann Arbor, MI) to generate consensus sequences from each isolate. These sequences have been deposited in GenBank under accession numbers (submission in progress). Further alignments and comparison to the reference sequence were performed using DNASTAR Lasergene 10 (DNASTAR Inc., Madison, WI). For NmE, we amplified and sequenced an 853bp fragment of *cseG* from 64 isolates as follows: 5 μL of lysate were amplified for 30 PCR cycles with 200 nM of each primer cpEgF15 (5’-TCTAAAATTGGCGGTTGAAA-3’) and cpEgR907 (5’-CTTTATTGGACTCTTTTACGACTA-3’), 25 μL 2x TaKaRa ExTaq PCR master mix in a 50-μL reaction. Each cycle included 10 s at 98°C, 30 s at 50°C and a 1 min extension step at 68°C. Resulting PCR products were sequenced as described above for the NmZ plasmids.

### Real-Time PCR Assay and Analysis

To evaluate the specificity of the primer-probe sets shown in Table 1, rt-PCR reactions were performed on strains from the proficiency panel under the following conditions: 25-μL reactions containing 400 nM of each primer, 200 nM of fluorescent probe, 12.5 μL of 2x QuantiTect Probe Kit (Qiagen) and 5 μL of DNA or lysate were assembled in MicroAmp Optical 96-Well Reaction Plates (Life Technologies). Reactions were run in an ABI Real-Time PCR System 7500 (Life Technologies) using these cycling parameters: 15 min at 95°C, followed by 40 cycles of 30 s at 95°C and 1 min at 60°C. The analysis performed by the 7500 System SDS Software v1.3.1 generated the threshold cycle (Ct) for each reaction. The passing criteria for primer-probe sets designed to identify Nm and each serogroup was to generate a Ct value appropriate to the number of copies added to the reaction and no Ct values with DNA template from other species. Primer-probe sets resulting in Ct values lower than 40 with any strain other than Nm were deemed nonspecific and therefore rejected as meningococcal identification sets.

To evaluate the testing panel of carriage isolates, rt-PCR reactions were assembled using 400 nM of each primer and 200 nM of probe, 12.5 μL of QuantiFast Probe PCR mix (Qiagen) and 5 μL of lysate in MicroAmp Optical 96-Well Reaction Plates. Reactions were performed under fast cycling conditions: 3 min at 95°C followed by 40 cycles of 3 s at 95°C and 35 s at 60°C. Each assay plate included negative control wells with no template and wells with ~1x10^4^ copies of a positive control plasmid carrying the target gene. Each of the eight genogrouping assays was performed as singleplex reactions. All reactions resulting in a Ct greater than 35 were designated as negative, because this value is approaching the limit of detection (LOD) of the assay. If the Ct value was lower than 30, the reaction was considered positive. Genogrouping reactions with Ct values between 30 and 35 were compared to the Ct obtained for *porA* with the same isolate and the difference between the two values was then calculated. If the Ct differential was greater than six (indicative of a near 80-fold difference in DNA input), the reaction was considered negative. Conversely, if this Ct difference was smaller than six, the reaction was considered positive. This algorithm was envisioned because all targets are present in one copy per genome and therefore each individual Nm test isolate was expected to yield similar Ct values with *porA*, *ctrA*, and the capsule grouping assay. The same algorithm was able to resolve reactions with positive results for two or more genogroups (Multiple, Table 2).

**Table 2:** Genogroup Distribution and Meningococcal Identification of the 234 Carriage Isolates by Real-Time PCR.

Serogroup	SASG	RT-PCR w/Algorithm	RT-PCR:SASGAgreement	*porA*	*ctrA*	*fhbp*	*porA*:*fhbp* ratio (%)
**B**	41	77	40/41	77	72	77	100
**C**	3	20	3/3	20	20	20	100
**E**	2	21	2/2	21	21	21	100
**W**	12	28	11/12	28	28	28	100
**X**	3	2	1/3	2	2	2	100
**Y**	6	27	4/6	27	27	27	100
**Z**		4		4	4	4	100
**Multiple** ^a^		2		2	2	2	
**Subtotal**	67	181	61/67 (91%)	181	176	181	100
***cnl***	n.a.	33		33	4	33	
**UKN**	n.a.	20		20	12	20	
**NG/NGG**	167	53	50/53	53	16	53	100
**Total**	234	234		234	193	234	100

n.a. = not applicable; NG = nongroupable by SASG assay; NGG = nongenogroupable by rt-PCR assays; UKN = unknown (no result by genogrouping rt-PCR assays and *cnl* PCR negative or not done).

^a^Positive result with two rt-PCR genogrouping assays (Ct<30).

Isolates with negative genogrouping results (nongenogroupable, NGG) were subject to PCR to determine the capsule null locus (*cnl*) as described by Claus et al. [20].

### SASG Assay

Serogrouping of the carriage isolate collection was accomplished by conducting slide agglutination with commercial antisera using published methods [16].

### fHbp Analysis

Characterizaton of fHbp variants in the carriage isolates was performed by PCR amplification and sequencing of *fhbp* as described in Murphy et al. [12]. fHbp variants segregate into two subfamilies, designated A and B; fHbp variant A05 corresponds to variant 5 from subfamily A.

## Results

### Real-time Universal PCR assays Specific for *N*. *meningitidis*

To develop rt-PCR assays for the unambiguous identification of Nm isolates, primer-probe sets were designed to target 16S rRNA, *crgA*, *porA*, or *ctrA* genes. For each gene we chose the primer-probe set displaying the highest efficiency and a sensitivity to detect as few as 100 genome copies. The specificity of the primer-probe sets was evaluated using a subset of 46 bacterial strains from the proficiency panel that included one NmC, one NmY, six Nm *cnl*, and the 38 additional non-meningococcal isolates (as detailed in Methods). Only the *porA* and *ctrA* assays were 100% specific for Nm (S1 Table). Although *N*. *subflava* contains *ctrA* [41], sequence polymorphisms do not permit annealing of these primers and probe [42]. As expected, the *ctrA* assay was positive with encapsulated meningococcal isolates and negative with the six isolates designated as *cnl* strains using the *cnl* PCR primers [20]. The 16S rRNA assay was positive for all *Neisseria spp*., making it unsuitable for the specific identification of Nm. Similarly, the *crgA* primer-probe set recognized *N*. *sicca* in addition to Nm and was therefore rejected as a meningococcal identification assay reagent. In summary, only the *porA* assay was sufficiently specific to identify Nm isolates in the proficiency panel. With the exception of *cnl* isolates, the assay targeting *ctrA* was also suitable for specific identification of Nm.

### *porA* and *ctrA* grouping of carriage strains by real-time PCR

After establishing that only the *porA* and *ctrA* probes were specific for Nm, they were tested using the Nm carriage isolate collection (*n* = 234). The *porA* real time assay was positive for all isolates. Using the *ctrA* rt-PCR assay, 193 of 234 isolates (82.5%) were *ctrA* positive and 42 were *ctrA* negative (Table 2 and S2 Table). The role of the *ctrA* assay in our scheme served two purposes. First, a *ctrA* positive result reinforced a positive identification of Nm accomplished by a *porA* positive result or acted as the sole Nm identifier in the event an isolate was *porA* negative. Secondly, a *ctrA* negative result was indicative of either sequence polymorphism (eg, as with *N*. *subflava*), or partial or complete deletion of the capsule operon. Although this is rare for invasive disease isolates, it is not uncommon in carriage isolates [20,43]. We therefore determined the number of *porA* positive, *ctrA* negative isolates that lacked the capsule biosynthesis and transport genes (*cnl*) among the collection of carriage isolates. Of the 42 isolates that tested negative for *ctrA*, 29 were confirmed to be *cnl* (69%).

### Identification of sequences that permit discrimination of NmZ and NmE

Following confirmation that the carriage isolates were meningococci using the porA and ctrA assays, group specific rt-PCR assays were required for serogroup assignment. While rt-PCR assays for serogroups A, B, C, W, X and Y are available (primer-probe placement illustrated in Fig 1), no rt-PCR assays for genogrouping NmE and NmZ have been reported. We first designed NmE and NmZ assays using sequences from the 5’-end of *ctrA* (Fig 1), but these lacked the required specificity. To select NmZ specific sequences we analysed genes within the capsule biosynthesis locus in this serogroup. The GenBank sequence (AJ744766) of the NmZ reference strain included 4 genes (*cszA*, *cszB*, *cszC* and *cszD*) in region A of the *cps* operon which were NmZ-specific. An alignment to determine the nucleotide similarity of this region in the eight NmZ isolates sequenced, revealed a 99% DNA sequence identity to the reference strain in the region harboring all four NmZ capsule biosynthesis genes (*cszA*, *cszB*, *cszC*, and *cszD*). Single nucleotide polymorphisms (SNPs) were identified in each gene, including single nucleotide deletions or insertions within *cszB*, *cszC*, and *cszD*. Isolate PMB3083 was found to carry the 845 bp insertion element *IS1301* at position 2909 of the *cszC* gene, which is commonly 3825 bp long. Five isolates had a *cszA* gene sequence identical to the reference and three isolates had only one nucleotide difference, making it the most conserved gene and a promising candidate for the design of a serogroup specific primer-probe set. A new primer-probe set targeting *cszA* was designed (Table 1, Fig 1) and 80 isolates (12 NmZ and 68 NmE) were evaluated by rt-PCR. All 12 NmZ isolates were correctly identified with the new primer-probe combination, while all 68 NmE isolates were PCR negative.

To select NmE specific sequences within the capsule biosynthesis locus we relied on the work reported by Zhu *et al* [44] and focused on *cseG*. We sequenced 853 bp of this gene in 64 out of 68 NmE isolates to design a novel primer-probe set (Table 1, Fig 1). The new NmE primer-probe combination was then used to evaluate the 80 NmE and NmZ isolates described above. All 68 NmE isolates were positive and the 12 NmZ isolates yielded negative results. In addition, the newly designed NmE and NmZ primer-probe combinations were only active against the respective NmE and NmZ meningococci when tested using a set of Nm strains that included representatives from each of the eight serogroups of interest. The NmE and NmZ reagents were also negative when tested against the 38 non-meningococcal isolates.

### Capsule grouping of carriage strains by real-time PCR

Initial evaluation of the genogrouping primers and probes with genomic DNA and lysates from meningococcal strains of the eight serogroups (A, B, C, E, W, X, Y and Z) demonstrated that they were serogroup-specific. They only identified isolates from the corresponding serogroup and none from the seven heterologous serogroups (S3 Table).

To genogroup the collection of carriage isolates (*n* = 234) by rt-PCR, reactions were run in singleplex format, with each 96-well reaction plate including a no template control and a positive control. Of the 234 carriage isolates in the collection, 181 isolates yielded positive results with at least one of the genogrouping assays and 53 isolates yielded negative results with all eight genogrouping assays (S2 Table). The genogroup distribution of these 181 genogroup positive Nm carriage isolates was NmB (77), NmC (20), NmE (21), NmW (28), NmX (2), NmY (27), NmZ (4), and mixed (2) (Table 2). None was positive for NmA. Twelve out of fourteen isolates with initially mixed results were resolved using the algorithm described in the Methods.

Of the 53 nongenogroupable (NGG) isolates, 29 (54.7%) were *cnl* and 37 (69.8%) were *ctrA* negative.

### Evaluation of the typing scheme in the context of carriage isolates

We comparatively evaluated the rt-PCR grouping results with the *porA*, *ctrA*, and *fhbp* assays. Every carriage isolate that was genogrouped by rt-PCR was also positive in assays that target genes coding for the two surface proteins, fHbp and PorA. The concordance between rt-PCR genogroup assignment and *ctrA* was lower, as five NmB isolates were *ctrA* negative (Table 2).

The SASG was also conducted to further assess the accuracy of the assay, although it is well documented that a large proportion of carriage strains are not groupable by SASG due to the lack of capsule expression [21]. A total of 67 isolates were serogrouped (S2 Table). The concordance between the molecular assay was compared to the SASG assay and it was determined that of the 67 isolates successfully grouped by SASG, 61 were grouped identically by rt-PCR (91% agreement, Table 2). Three of the six dissenting isolates were nongenogroupable by rt-PCR and three classified to a different group. On the other hand, 167 out of 234 isolates in the collection were NG by SASG (Table 2). However, 112 of these isolates were genogrouped by the rt-PCR assays. A breakdown by serogroup and the agreement with rt-PCR is shown in Table 2. The genogrouping of Nm carriage isolates performed by these rt-PCR assays is clearly preferred to SASG not only because it is able to group more isolates but also because of its throughput, sensitivity, and simplicity.

### fHbp Composition and diversity in the carriage isolate collection

**Fig 2:**
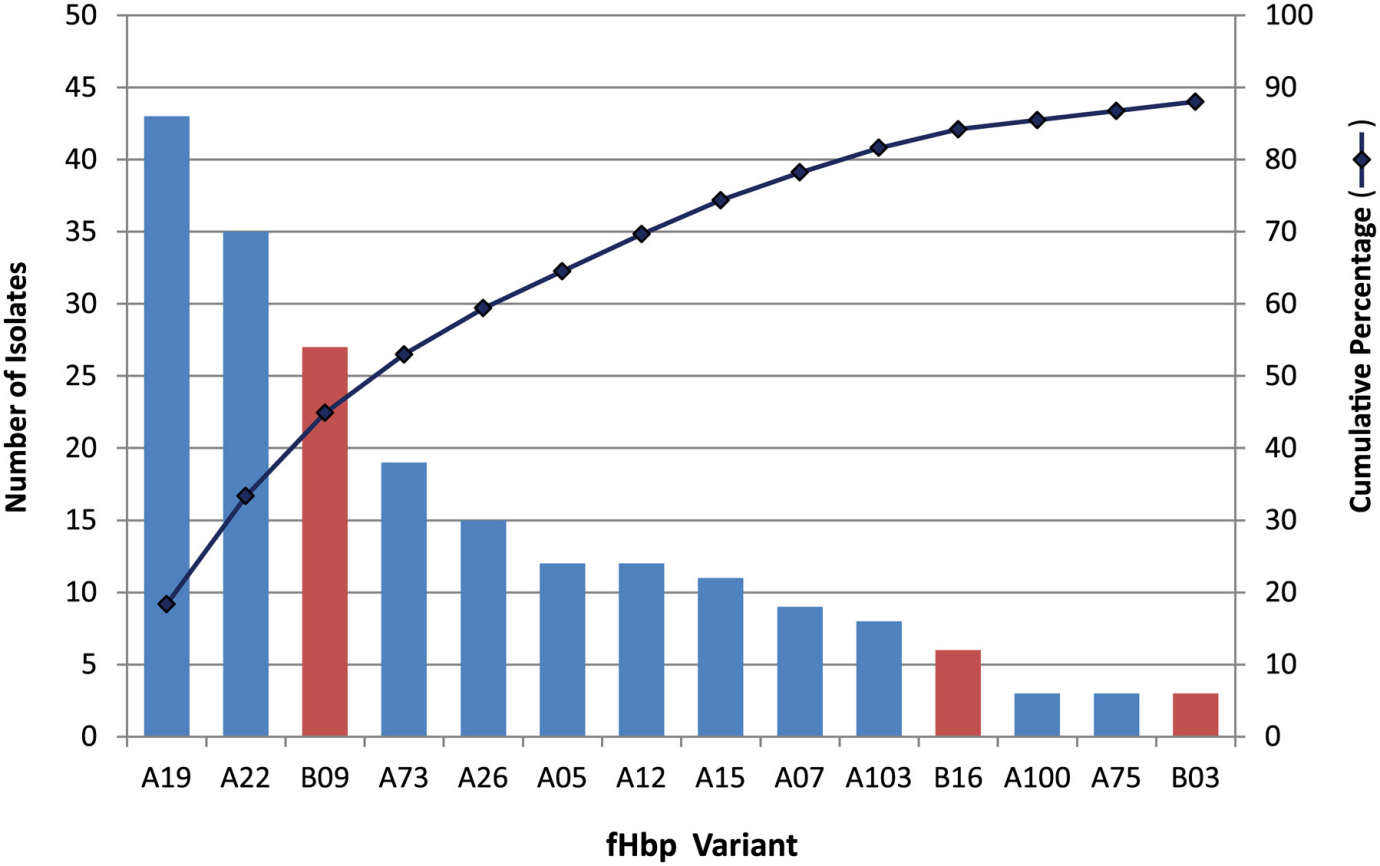
Diversity of the fHbp variants in the carriage isolate collection. Distribution of fHbp variants found in three or more isolates. Fourteen fHbp variants encompassed 206 (88%) isolates in the collection. Blue bars, fHbp subfamily A variants; red bars, fHbp subfamily B variants.

**Fig 3:**
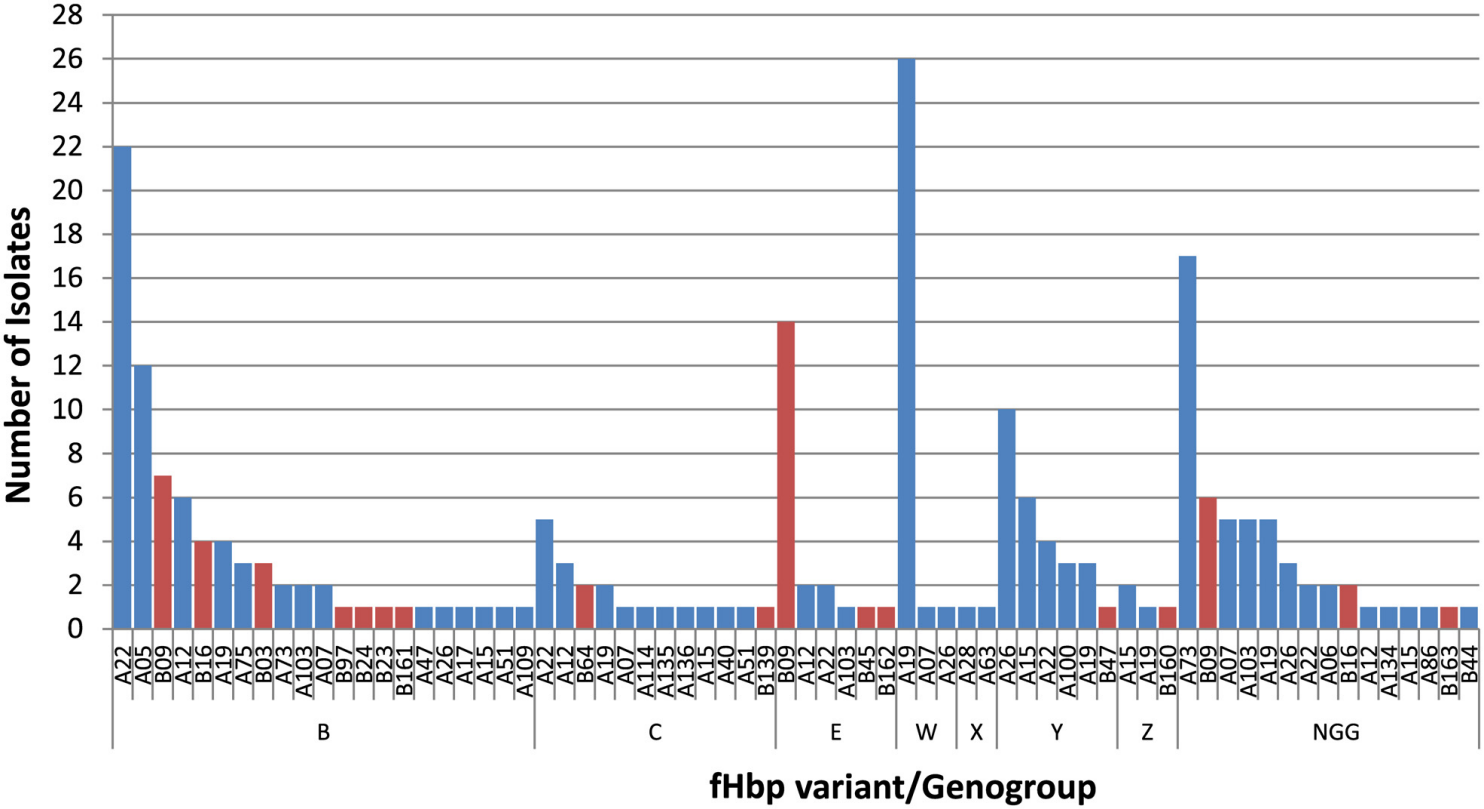
Distribution of the fHbp variants by genogroup in the carriage isolate collection. Carriage isolates were categorized by fHbp variant within each genogroup and are shown according to number of isolates identified. Blue bars, fHbp subfamily A variants; red bars, fHbp subfamily B variants. NGG, nongenogroupable by rt-PCR.

The carriage isolates were obtained from adolescents and young adults over a five-year period (1997–2001) that encompassed the introduction of NmC vaccines in the United Kingdom. The gene that encodes fHbp was detected in all 234 isolates. As has been reported in other Nm carriage collections [45,46], the majority of the isolates typed to fHbp subfamily A (185 or 79%). Fourteen fHbp variants, each identified in three or more isolates, together represented 88% of the isolates in the study (Fig 2). In this collection, a broader diversity of fHbp variant types were identified for genogroups B, C, E, and Z than for W, X, and Y, which were primarily composed of isolates carrying fHbp subfamily A variants (Fig 3). There were 21 different fHbp variants distributed among isolates genogrouped as NmB, with the most abundant being variant A22 (*n* = 22). NmW isolates were more homogeneous and predominantly A19 (*n* = 26). The distribution of variant B09 spanned genogroups B, E and NG, but was primarily found in isolates genogrouped as E (*n* = 14).

## Discussion

Reliable, sensitive and high-throughput assays allowing the identification and characterization of Nm isolates are necessary to study the impact of vaccination campaigns on meningococcal carriage. Here, we describe the development and evaluation of rt-PCR assays for both the detection and genogroup assignment for Nm.

Conventional PCR has been described as a successful approach by several groups for the identification of Nm disease-causing strains [25,27,44,47,48]. Typing of carriage isolates is considerably more complex. Nm is naturally competent and undergoes rapid genetic changes [49]. While the human pharynx is the reservoir for Nm, disease isolates from within geographic regions tend to be from a limited range of clonal complexes [30]. The majority of carriage strains do not express capsule due to either phase variation [50] or the lack of an intact capsule locus [20]. Although unencapsulated strains of Nm are not generally associated with invasive disease, in rare instances *cnl* strains have been isolated from subjects with meningococcal disease [51]. Assays are based around invasive isolates and though it is assumed that the same approaches will work in carriage, neither approaches tested here were able to work for all isolates, even in the presence of *ctrA*, most likely due to small genetic changes. Strains are genetically competent so even seemingly harmless capsule-deficient strains can recombine to become virulent [52]; therefore, a vaccine that targets protein antigens may have utility in preventing the spread of these harmless isolates, too.

Clinical identification of Nm by rt-PCR has been accomplished by targeting various sequences, including 16S rRNA, insertion elements, *crgA*, *ctrA*, *porA*, and most recently *sodC* [29]. Although the *sodC* assay has been shown to be very robust for detection of Nm, it was not evaluated here because it was published after this study had been completed. Our evaluation indicated that rt-PCR assays based on *porA* and *ctrA* are specific and accurate enough to identify Nm among other oropharyngeal associated bacteria. Meningococcal identification based on conserved regions of *porA* has been described before by Whiley [31] and Jordens [28] who both developed *porA* rt-PCR assays. Some reports described NmB and NmC strains lacking the *porA* gene [53–55], but this appears to be uncommon. Harrison et al. [55] reported that about 13% of the invasive NmC strains in their study had the *porA* gene deleted. We hypothesized that by including a redundant assay such as one targeting *ctrA*, the number of false negative results produced by *porA* minus isolates could be reduced. However, the *porA* assay by itself appears to be robust for identifying Nm, judged by the positive results in all 234 meningococcal carriage isolates tested. In contrast, the assay targeting *ctrA* returned negative results in 42 strains, of which 29 were found to lack all the capsule biosynthesis and transport genes (*cnl*), which was in agreement with previous observations [20,21]. In our panel, 69% of the *ctrA* negative isolates were also *cnl* and only 11.9% (5/42) carried at least a portion of the capsule locus since they were classified as NmB (rt-PCR positive at *csb*). The negative *ctrA* results observed in the other eight isolates (classified as NGG) may be caused by other rearrangement events [21]. Although we specifically designed the *ctrA* assay to target a conserved region of the gene, we cannot exclude the possibility that sequence polymorphisms resulted in some *ctrA*-containing strains being identified as *ctrA*-negative in our assay. For instance, in 2010, two laboratories independently reported invasive Nm strains harboring *ctrA* variants that were missed by the *ctrA* rt-PCR assay routinely used as a confirmatory test in hospital settings [32,33]; our *ctrA* primers and probe were specifically designed to capture these novel *ctrA* variants, if present.

All 234 isolates in the testing panel carried the gene encoding the outer membrane lipoprotein fHbp, which is the antigen in the NmB vaccine Trumenba and one of the component antigens in Bexsero. Although Trumenba is made up of lipidated fHbp variants A05 and B01, immune sera have bactericidal activity against strains expressing heterologous variants in nonclinical [13] and Phase 1/2 clinical studies [56]. Sequence typing of the carriage isolates classified fHbp mostly to subfamily A (185 isolates). Counting of fHbp variants found in three or more isolates resulted in 14 different variants that together make up 88% of the panel of 234 isolates. Although *fhbp* was present in isolates from all eight serogroups, we predict that its inherent nucleotide sequence diversity will prevent the design of a universal diagnostic rt-PCR assay.

Also, in this study we developed rt-PCR assays with the goal of identifying up to eight (A, B, C, E, W, X, Y, and Z) distinct Nm capsular polysaccharide groups. Nucleotide sequences encoding capsule biosynthesis genes from serogroups A, B, C, W, X and Y, unlike E and Z, were readily available in GenBank. Evaluation of the testing panel with initial primers-probe sets that genogroup NmE and NmZ, based on the *ctrA* gene, revealed random cross-reactivity with isolates of other serogroups. Because of this observation and the likelihood of missing prospective *ctrA* negative isolates, they were discontinued. The choice of *ctrA* as a gene for grouping NmE and NmZ described by others [40,48] was prompted by the limited availability of sequences encoding their capsule biosynthesis genes, a hurdle to confidently design primer-probes targeting these genes. To further optimize these two assays, we sequenced the capsule loci of NmE and NmZ strains and designed specific primers and probes based on capsule biosynthesis genes unique to each serogroup (*cseG* and *cszA*, respectively). We sequenced *cseG* in 64 NmE isolates to get consensus regions for primer design. The *cszA* gene was selected based on the results from sequencing region A of the *cps* locus in eight NmZ isolates. One of these harbored the insertion element IS*1301* within *cszC*. Our redesigned primers and probes targeting the capsular biosynthesis genes increased the specificity of the genogrouping assays for serogroups E and Z. An alignment of our *cszA* rt-PCR amplicon with the *cshA* gene in the serogroup H strain previously reported [35] found 100% homology to the probe and one mismatch in each of the forward and reverse primers. However, lack of NmH isolates prevented us from evaluating the cross-reactivity of the NmZ sets with NmH. In the future, a reassessment of this primer-probe set will be facilitated by the availability of sequence data from additional NmH isolates.

The eight rt-PCR assays were employed to genogroup a diverse set of 234 meningococcal carriage isolates. They genogrouped 179 of them, 112 more than grouped by SASG. Among the isolates serogrouped by SASG, there was a 91% (61/67) agreement with the rt-PCR method. By rt-PCR, 77 isolates were classified as NmB, compared with the 41 serogrouped as NmB by SASG. NmW (28) and NmY (27) were followed by a surprisingly high number of NmE isolates (21) in comparison to the number of NmC (20), NmZ (4), and NmX (2) isolates. As expected, no NmA was found among these European isolates.

The combined speciation and capsule genogrouping assays were found to be specific and accurate in identifying and classifying Nm. PCR methods for genogrouping have inherent limitations. As an illustration, not only do they have a narrow range of tolerance to polymorphisms, but they are unable to determine the underlying mechanism behind a nongroupable (NGG) result. As bacterial whole genome sequence (WGS) methods and analysis pipelines become more efficient, the basis for these inherent limitations will become increasingly apparent. WGS data will also have utility in characterizing carriage isolates and teaching us more about genetic exchanges and rearrangements in this complex and dangerous organism. Its inherent higher resolution could help explain the phenotypic differences observed among isolates. Nonetheless, for fast identification of meningococci and the genogrouping of carriage isolates, the rt-PCR assay is the current method of choice because of its speed, simplicity, and higher throughput.

In summary, the data presented here show that the rt-PCR assays developed for identification and genogrouping of eight Nm serogroups (A, B, C, E, W, X, Y, and Z) are specific and sensitive and have been implemented for the survey of meningococcal carriage in healthy subjects.

## Supporting Information

S1 Table Ct values obtained with 16S, *crgA*, *porA*, and *ctrA* assays in a bacterial panel of 46 strains. (PDF)

S2 Table Serogroup, genogroup and Ct values of 10 rt-PCR assays in 234 carriage isolates. (PDF)

S3 Table Specificity of meningococcal genogrouping assays. (PDF)
