# Supplementary material for: Optimization of Molecular Approaches to Genogroup Neisseria meningitidis Carriage Isolates and Implications for Monitoring the Impact of New Serogroup B Vaccines
Source: PLoS One. 2015 Jul 6;10(7):e0132140. doi: 10.1371/journal.pone.0132140 (PMC4493136; doi:10.1371/journal.pone.0132140)
Supplement: S1 Table — (PDF) [file pone.0132140.s001.pdf]

S1 Table. Threshold Cycle (Ct) values obtained with 16S rRNA, *crgA*, *porA*, and *ctrA* primers and probes in a bacterial panel of 46 strains. Only *porA* and *ctrA* are specific for *N. meningitidis*.

Strain	16S rRNA	*crgA*	*porA*	*ctrA*
N. meningitidis C PMB1890	23.10	22.89	23.67	21.81
N. meningitidis Y PMB1063	13.39	14.11	15.55	13.83
N. meningitidis cnl PMB221	ND	ND	25.10	No Ct
N. meningitidis cnl PMB2380	ND	ND	21.73	No Ct
N. meningitidis cnl PMB1525	ND	ND	20.31	No Ct
N. meningitidis cnl PMB1345	ND	ND	16.77	No Ct
N. meningitidis cnl PMB976	ND	ND	20.08	No Ct
N. meningitidis cnl PMB1462	ND	ND	19.75	No Ct
N. cinerea ATCC 14685	20.86	No Ct	No Ct	No Ct
N. gonorrhoeae ATCC 27628	No Ct	No Ct	No Ct	No Ct
N. gonorrhoeae ATCC 49498	No Ct	No Ct	No Ct	No Ct
N. lactamica ATCC 23971	No Ct	No Ct	No Ct	No Ct
N. lactamica ATCC 49142	No Ct	No Ct	No Ct	No Ct
N. sicca ATCC 29256	20.98	No Ct	No Ct	No Ct
N. sicca ATCC 25259	17.57	25.85	No Ct	No Ct
N. subflava ATCC 19243	22.33	No Ct	No Ct	No Ct
N. subflava ATCC 49275	16.25	No Ct	No Ct	No Ct
H. aegyptus ATCC 43790	No Ct	No Ct	No Ct	No Ct
H. haemolyticus ATCC 33390	No Ct	No Ct	No Ct	No Ct
H. influenzae non-type ATCC 8149	No Ct	No Ct	No Ct	No Ct
H. influenzae non-type ATCC 9333	No Ct	No Ct	No Ct	No Ct
H. influenzae type a ATCC 9006	No Ct	No Ct	No Ct	No Ct
H. influenzae type b ATCC 9795	No Ct	No Ct	No Ct	No Ct
H. influenzae type b ATCC 10211	No Ct	No Ct	No Ct	No Ct
H. influenzae type c ATCC 9007	No Ct	No Ct	No Ct	No Ct
H. influenzae type c ATCC 45690	No Ct	No Ct	No Ct	No Ct
H. influenzae type d ATCC 9332	No Ct	No Ct	No Ct	No Ct
H. influenzae type e ATCC 8142	No Ct	No Ct	No Ct	No Ct
H. influenzae type f ATCC 9833	No Ct	No Ct	No Ct	No Ct
H. influenzae type i ATCC 49144	No Ct	No Ct	No Ct	No Ct
H. parainfluenzae ATCC 33392	No Ct	No Ct	No Ct	No Ct
H. parainfluenzae ATCC 33966	No Ct	No Ct	No Ct	No Ct
S. agalactiae GAR54	ND	ND	No Ct	No Ct
S. agalactiae GAR154	ND	ND	No Ct	No Ct
S. pneumoniae type 1	ND	ND	No Ct	No Ct
S. pneumoniae type 5	ND	ND	No Ct	No Ct
S. pneumoniae type 14	ND	ND	No Ct	No Ct

**Strain**	**16S rRNA**	***crgA***	***porA***	***ctrA***
S. pneumoniae type 19F	ND	ND	No Ct	No Ct
S. pyogenes SF370	ND	ND	No Ct	No Ct
S. aureus 397	ND	ND	No Ct	No Ct
S. aureus 659-018	ND	ND	No Ct	No Ct
M. catarrhalis 035E	ND	ND	No Ct	No Ct
M. catarrhalis ATCC 25240	ND	ND	No Ct	No Ct
M. catarrhalis ATCC 43617	ND	ND	No Ct	No Ct
M. catarrhalis ATCC 43618	ND	ND	No Ct	No Ct
M. catarrhalis SH5	ND	ND	No Ct	No Ct

ND= assay not performed
Ct values represent the average of two replicates
