# Supplementary material for: Optimization of Molecular Approaches to Genogroup Neisseria meningitidis Carriage Isolates and Implications for Monitoring the Impact of New Serogroup B Vaccines
Source: PLoS One. 2015 Jul 6;10(7):e0132140. doi: 10.1371/journal.pone.0132140 (PMC4493136; doi:10.1371/journal.pone.0132140)
Supplement: S2 Table — (PDF) [file pone.0132140.s002.pdf]

S2 Table. Serogroup, genogroup and Ct values of *porA*, *ctrA* and eight genogrouping rt-PCR assays in 234 carriage isolates.

Strain	Group (SASG)	GGroup rt-PCR	cnl PCR	porA Ct	ctrA Ct	NmA Ct	NmB Ct	NmC Ct	NmW Ct	NmX Ct	NmY Ct	NmE Ct*	NmE Ct	NmZ Ct*	NmZ Ct	porA -ctrA	Delta Ct
PMB1475	NG	NGG	*cnl*	24.18	No Ct	No Ct	No Ct	No Ct	No Ct	No Ct	No Ct	39.26	ND	No Ct	ND	NA	
PMB3421	NG	NGG	*cnl*	20.75	No Ct	No Ct	No Ct	No Ct	No Ct	No Ct	No Ct	No Ct	ND	No Ct	ND	NA	
PMB995	NG	NGG	*cnl*	26.60	No Ct	No Ct	No Ct	No Ct	No Ct	No Ct	No Ct	No Ct	ND	No Ct	ND	NA	
PMB643	NG	B		14.92	15.08	No Ct	29.93	No Ct	No Ct	No Ct	34.54	No Ct	ND	No Ct	ND	0.2	19.6
PMB3316	NG	NGG	*cnl*	16.03	16.38	No Ct	No Ct	No Ct	No Ct	No Ct	No Ct	No Ct	ND	No Ct	ND	0.3	
PMB1525	NG	NGG	*cnl*	23.98	34.10	No Ct	No Ct	No Ct	No Ct	No Ct	No Ct	37.24	ND	No Ct	ND	10.1	
PMB1345	NG	NGG	*cnl*	23.02	No Ct	No Ct	No Ct	No Ct	No Ct	No Ct	No Ct	37.57	ND	No Ct	ND	NA	
PMB1462	NG	NGG	*cnl*	21.56	39.18	No Ct	No Ct	No Ct	38.76	No Ct	No Ct	No Ct	ND	No Ct	ND	17.6	
PMB2540	NG	B	*cnl*	19.56	34.16	No Ct	20.72	No Ct	No Ct	No Ct	No Ct	30.58	36	No Ct	ND	14.6	
PMB1255	NG	NGG	*cnl*	19.64	No Ct	No Ct	33.87	No Ct	No Ct	No Ct	No Ct	No Ct	ND	No Ct	ND	NA	14.2
PMB439	NG	NGG	*cnl*	20.11	No Ct	No Ct	No Ct	No Ct	No Ct	No Ct	No Ct	No Ct	ND	No Ct	ND	NA	
PMB3085	NG	NGG	*cnl*	19.21	No Ct	No Ct	No Ct	No Ct	No Ct	No Ct	No Ct	38.07	ND	No Ct	ND	NA	
PMB971	NG	NGG	*cnl*	19.75	No Ct	No Ct	No Ct	No Ct	No Ct	No Ct	No Ct	No Ct	ND	No Ct	ND	NA	
PMB1680	NG	NGG	*cnl*	24.50	No Ct	No Ct	No Ct	No Ct	No Ct	No Ct	No Ct	No Ct	ND	No Ct	ND	NA	
PMB3220	NG	NGG	*cnl*	19.98	No Ct	No Ct	No Ct	No Ct	No Ct	No Ct	No Ct	38.6	ND	No Ct	ND	NA	
PMB2252	NG	NGG	*cnl*	18.42	No Ct	No Ct	No Ct	No Ct	No Ct	No Ct	No Ct	No Ct	ND	No Ct	ND	NA	
PMB3280	NG	NGG	*cnl*	24.09	32.83	No Ct	38.65	No Ct	No Ct	No Ct	No Ct	No Ct	ND	No Ct	ND	8.7	
PMB2080	NG	NGG	*cnl*	22.00	33.83	No Ct	32.86	No Ct	No Ct	No Ct	No Ct	No Ct	ND	No Ct	ND	11.8	10.9
PMB976	NG	NGG	*cnl*	21.14	No Ct	No Ct	No Ct	No Ct	No Ct	No Ct	No Ct	37.53	No Ct	No Ct	ND	NA	
PMB1197	NG	NGG	*cnl*	21.72	33.54	No Ct	No Ct	No Ct	No Ct	No Ct	No Ct	33.48	No Ct	No Ct	ND	11.8	11.8
PMB1980	NG	NGG	*cnl*	18.67	26.18	No Ct	39.18	No Ct	No Ct	No Ct	No Ct	No Ct	ND	No Ct	ND	7.5	
PMB1484	NG	NGG	*cnl*	19.78	30.26	No Ct	No Ct	No Ct	No Ct	No Ct	No Ct	38.35	No Ct	No Ct	ND	10.5	
PMB3289	NG	NGG	*cnl*	17.52	32.88	No Ct	No Ct	No Ct	No Ct	No Ct	No Ct	30.12	ND	No Ct	ND	15.4	12.6
PMB2108	NG	NGG	*cnl*	20.09	31.69	No Ct	No Ct	No Ct	38.55	No Ct	No Ct	No Ct	ND	No Ct	ND	11.6	
PMB1218	NG	NGG	*cnl*	18.53	No Ct	No Ct	No Ct	No Ct	No Ct	No Ct	No Ct	No Ct	ND	No Ct	ND	NA	
PMB2332	NG	NGG	*cnl*	19.65	No Ct	No Ct	No Ct	No Ct	No Ct	No Ct	No Ct	No Ct	ND	No Ct	ND	NA	
PMB2793	NG	NGG	*cnl*	20.49	36.02	No Ct	39.37	No Ct	37.72	No Ct	No Ct	No Ct	ND	No Ct	ND	15.5	
PMB1750	NG	NGG	*cnl*	23.49	34.30	No Ct	33.72	No Ct	No Ct	No Ct	No Ct	No Ct	ND	No Ct	ND	10.8	10.2

Strain	Group (SASG)	GGroup rt-PCR	cnl PCR	porA Ct	ctrA Ct	NmA Ct	NmB Ct	NmC Ct	NmW Ct	NmX Ct	NmY Ct	NmE Ct*	NmE Ct	NmZ Ct*	NmZ Ct	porA -ctrA	Delta Ct
PMB3519	NG	NGG	*cnl*	21.42	32.31	No Ct	38.44	No Ct	No Ct	No Ct	37.82	30.65	ND	No Ct	ND	10.9	9.2
PMB1383	NG	NGG	*cnl*	22.26	32.75	No Ct	32.08	37.45	No Ct	No Ct	No Ct	36.8	ND	No Ct	ND	10.5	9.8
PMB347	NG	B	*cnl*	26.08	30.15	No Ct	29.47	No Ct	No Ct	No Ct	No Ct	No Ct	ND	No Ct	ND	4.1	
PMB2708	NG	NGG	*cnl*	21.62	26.38	No Ct	39.29	No Ct	No Ct	No Ct	No Ct	No Ct	ND	No Ct	ND	4.8	
PMB1353	NG	NGG	*cnl*	20.69	30.62	No Ct	No Ct	No Ct	36.15	No Ct	No Ct	37.74	ND	37.55	ND	9.9	
PMB2285	NG	NGG	*cnl*	21.36	30.80	No Ct	No Ct	No Ct	No Ct	No Ct	No Ct	No Ct	ND	No Ct	ND	9.4	
PMB3162	NG	NGG	*cnl*	21.03	28.65	No Ct	No Ct	No Ct	No Ct	No Ct	No Ct	No Ct	ND	No Ct	ND	7.6	
PMB1141	NG	C		13.95	29.30	No Ct	33.86	22.04	No Ct	No Ct	No Ct	38.68	ND	No Ct	ND	15.4	8.1
PMB1946	NG	NGG	*cnl*	21.35	31.10	No Ct	No Ct	No Ct	No Ct	No Ct	No Ct	37.52	ND	No Ct	ND	9.7	
PMB242	NG	NGG	ND	19.32	No Ct	No Ct	No Ct	No Ct	No Ct	No Ct	No Ct	35.15	No Ct	No Ct	No Ct	NA	
PMB1104	Y	NGG	ND	22.31	22.20	No Ct	No Ct	No Ct	No Ct	No Ct	32.67	No Ct	ND	No Ct	ND	0.1	10.4
PMB1741	NG	B	ND	17.65	No Ct	No Ct	16.44	No Ct	No Ct	32.59	No Ct	No Ct	ND	No Ct	ND	NA	14.9
PMB1088	W	NGG	ND	18.79	31.71	No Ct	No Ct	No Ct	No Ct	No Ct	No Ct	No Ct	ND	No Ct	ND	12.9	
PMB3642	B	NGG	ND	17.92	24.59	No Ct	36	No Ct	30.87	No Ct	No Ct	38.56	ND	No Ct	ND	6.7	13.0
PMB968	NG	B	No *cnl*	21.67	32.42	No Ct	17.37	No Ct	No Ct	No Ct	No Ct	No Ct	ND	No Ct	ND	10.7	
PMB1567	NG	NGG	NR	20.55	No Ct	No Ct	No Ct	No Ct	No Ct	No Ct	No Ct	No Ct	ND	No Ct	ND	NA	
PMB2616	NG	NGG	NR	15.39	15.48	No Ct	35.12	37.12	No Ct	No Ct	No Ct	33.77	ND	No Ct	ND	0.1	18.4
PMB1934	NG	NGG	NR	22.18	21.22	No Ct	32.69	38.24	No Ct	No Ct	No Ct	34.79	ND	No Ct	ND	1.0	10.5
PMB419	NG	NGG	NR	20.22	22.58	No Ct	No Ct	No Ct	No Ct	No Ct	No Ct	No Ct	ND	No Ct	ND	2.4	
PMB2638	NG	NGG	NR	19.49	19.20	No Ct	No Ct	33	No Ct	No Ct	No Ct	No Ct	ND	No Ct	ND	0.3	13.5
PMB804	NG	NGG	NR	18.79	No Ct	No Ct	No Ct	No Ct	No Ct	No Ct	No Ct	No Ct	ND	No Ct	ND	NA	
PMB312	NG	NGG	NR	18.81	33.17	No Ct	No Ct	No Ct	No Ct	No Ct	No Ct	38.64	ND	No Ct	ND	14.4	
PMB3474	NG	NGG	NR	18.77	18.80	No Ct	No Ct	No Ct	No Ct	No Ct	No Ct	No Ct	ND	No Ct	ND	0.0	
PMB2759	NG	NGG	NR	20.84	20.73	No Ct	No Ct	No Ct	No Ct	No Ct	No Ct	37.39	No Ct	No Ct	ND	0.1	
PMB2084	NG	NGG	NR	18.13	No Ct	No Ct	No Ct	No Ct	No Ct	No Ct	No Ct	37.74	ND	No Ct	ND	NA	
PMB1668	NG	NGG	NR	23.65	39.11	No Ct	No Ct	No Ct	38.66	No Ct	No Ct	No Ct	ND	No Ct	ND	15.5	
PMB2581	NG	NGG	NR	22.37	21.62	No Ct	36.22	No Ct	No Ct	No Ct	No Ct	No Ct	ND	No Ct	ND	0.8	
PMB1990	NG	E		19.32	20.84	No Ct	No Ct	No Ct	39.53	No Ct	No Ct	No Ct	18.12	No Ct	ND	1.5	
PMB3412	NG	NGG	NR	24.33	24.16	No Ct	No Ct	No Ct	No Ct	No Ct	No Ct	No Ct	ND	No Ct	ND	0.2	

Strain	Group (SASG)	GGroup rt-PCR	cnl PCR	porA Ct	ctrA Ct	NmA Ct	NmB Ct	NmC Ct	NmW Ct	NmX Ct	NmY Ct	NmE Ct*	NmE Ct	NmZ Ct*	NmZ Ct	porA -ctrA	Delta Ct
PMB460	NG	NGG	NR	20.72	20.83	No Ct	No Ct	No Ct	No Ct	No Ct	No Ct	No Ct	ND	No Ct	ND	0.1	
PMB2548	NG	Y		20.40	20.45	No Ct	37.24	No Ct	No Ct	No Ct	28.8	35.07	ND	No Ct	ND	0.1	
PMB1781	NG	NGG	NR	22.47	32.37	No Ct	38.21	39.41	No Ct	No Ct	No Ct	No Ct	ND	No Ct	ND	9.9	
PMB2574	NG	NGG	NR	18.87	18.62	No Ct	32.95	No Ct	No Ct	No Ct	No Ct	No Ct	ND	No Ct	ND	0.2	14.1
PMB3507	NG	Y		24.96	24.65	No Ct	No Ct	No Ct	No Ct	No Ct	26.1	No Ct	ND	No Ct	ND	0.3	
PMB1155	B	B		16.99	16.49	No Ct	15.66	No Ct	No Ct	No Ct	No Ct	No Ct	ND	No Ct	ND	0.5	
PMB473	B	B		21.82	21.66	No Ct	22.91	No Ct	No Ct	No Ct	No Ct	No Ct	ND	No Ct	ND	0.2	
PMB3509	B	B		24.91	24.75	No Ct	30.65	No Ct	No Ct	No Ct	No Ct	No Ct	ND	No Ct	ND	0.2	5.7
PMB1827	NG	Y		26.90	26.84	No Ct	No Ct	No Ct	No Ct	No Ct	29.09	No Ct	ND	No Ct	ND	0.1	
PMB2333	B	B		18.93	19.00	No Ct	18.89	No Ct	No Ct	No Ct	No Ct	36.42	No Ct	No Ct	No Ct	0.1	
PMB724	B	B		22.24	21.70	No Ct	23.26	No Ct	No Ct	No Ct	No Ct	No Ct	ND	No Ct	ND	0.5	
PMB3011	X	B		19.25	18.88	No Ct	18.66	No Ct	No Ct	No Ct	No Ct	No Ct	ND	No Ct	ND	0.4	
PMB2061	B	B		21.75	21.70	No Ct	20.74	No Ct	No Ct	No Ct	No Ct	No Ct	ND	No Ct	ND	0.1	
PMB2338	W	W		15.72	14.96	No Ct	36.67	No Ct	14.12	No Ct	28.3	No Ct	ND	No Ct	ND	0.8	12.6
PMB1821	NG	Y		19.98	19.86	No Ct	No Ct	No Ct	No Ct	No Ct	21.23	No Ct	ND	No Ct	ND	0.1	
PMB3147	B	B		15.57	15.26	No Ct	15.37	No Ct	No Ct	No Ct	No Ct	No Ct	ND	No Ct	ND	0.3	
PMB676	B	B		15.90	15.56	No Ct	15.55	No Ct	No Ct	No Ct	No Ct	No Ct	ND	No Ct	ND	0.3	
PMB1742	B	B		16.35	16.31	No Ct	16.83	No Ct	No Ct	No Ct	No Ct	No Ct	ND	No Ct	ND	0.0	
PMB1358	NG	C		19.36	19.04	No Ct	No Ct	19.86	No Ct	No Ct	No Ct	No Ct	ND	38.91	ND	0.3	
PMB2109	NG	B		17.49	17.33	No Ct	17.32	No Ct	No Ct	No Ct	No Ct	No Ct	ND	No Ct	ND	0.2	
PMB2659	NG	B		19.10	18.82	No Ct	18.61	No Ct	No Ct	No Ct	No Ct	No Ct	ND	No Ct	ND	0.3	
PMB2758	B	B		15.00	14.97	No Ct	15.5	No Ct	No Ct	No Ct	No Ct	No Ct	ND	No Ct	ND	0.0	
PMB248	NG	B		19.64	19.44	No Ct	19.76	No Ct	No Ct	No Ct	No Ct	No Ct	ND	No Ct	ND	0.2	
PMB3399	NG	Y		16.78	16.96	No Ct	35.73	No Ct	37.35	No Ct	18.03	No Ct	ND	No Ct	ND	0.2	
PMB1465	NG	W		14.27	14.71	No Ct	36.06	No Ct	18.04	37.25	No Ct	No Ct	ND	No Ct	ND	0.4	
PMB1132	NG	W		22.78	22.54	No Ct	No Ct	39.38	20.62	No Ct	No Ct	No Ct	ND	No Ct	ND	0.2	
PMB2091	B	B		15.61	15.49	No Ct	15.16	No Ct	No Ct	No Ct	No Ct	No Ct	ND	No Ct	ND	0.1	
PMB3397	Y	Y		21.25	21.06	No Ct	33.76	No Ct	No Ct	No Ct	22.71	No Ct	ND	No Ct	ND	0.2	12.5
PMB1971	B	B		18.15	17.77	No Ct	16.97	No Ct	No Ct	No Ct	No Ct	No Ct	ND	No Ct	ND	0.4	

Strain	Group (SASG)	GGroup rt-PCR	cnl PCR	porA Ct	ctrA Ct	NmA Ct	NmB Ct	NmC Ct	NmW Ct	NmX Ct	NmY Ct	NmE Ct*	NmE Ct	NmZ Ct*	NmZ Ct	porA -ctrA	Delta Ct
PMB3076	NG	B		25.34	24.94	No Ct	18.04	No Ct	No Ct	No Ct	No Ct	No Ct	ND	No Ct	ND	0.4	
PMB3307	NG	B		14.10	23.74	No Ct	19.4	38.06	No Ct	No Ct	No Ct	No Ct	ND	No Ct	ND	9.6	
PMB2884	NG	Y		20.42	20.16	No Ct	36.86	No Ct	No Ct	No Ct	17.2	No Ct	ND	No Ct	ND	0.3	
PMB3057	NG	E		20.64	22.73	No Ct	28.14	No Ct	No Ct	No Ct	No Ct	16.89	14.21	No Ct	ND	2.1	7.5
PMB2947	B	B		15.74	15.70	No Ct	14.89	No Ct	No Ct	No Ct	No Ct	34.05	ND	No Ct	ND	0.0	18.3
PMB1078	NG	B		21.12	20.71	No Ct	18.05	No Ct	No Ct	No Ct	No Ct	No Ct	ND	No Ct	ND	0.4	
PMB818	E	E		17.01	16.77	No Ct	31.57	No Ct	No Ct	No Ct	No Ct	16.68	28.88	No Ct	ND	0.2	14.6
PMB2456	NG	E		23.38	25.42	No Ct	No Ct	No Ct	No Ct	No Ct	No Ct	20.45	19	No Ct	ND	2.0	
PMB2113	B	B		16.18	15.65	No Ct	15.33	No Ct	No Ct	No Ct	No Ct	No Ct	ND	No Ct	ND	0.5	
PMB1595	B	B		17.28	16.93	No Ct	16.29	No Ct	No Ct	35.88	No Ct	No Ct	ND	No Ct	ND	0.3	
PMB620	NG	Y		20.52	20.06	No Ct	39	No Ct	No Ct	No Ct	18.03	No Ct	ND	No Ct	ND	0.5	
PMB1075	NG	E		27.10	29.02	No Ct	No Ct	No Ct	No Ct	No Ct	No Ct	19.74	18.28	No Ct	ND	1.9	7.4
PMB203	C	C		15.56	15.34	No Ct	No Ct	14.32	No Ct	No Ct	No Ct	No Ct	ND	No Ct	ND	0.2	
PMB2807	B	B		18.03	18.27	No Ct	16.81	No Ct	No Ct	No Ct	No Ct	No Ct	ND	No Ct	ND	0.2	
PMB35	NG	E		21.14	21.09	No Ct	No Ct	No Ct	29.89	No Ct	No Ct	18.93	14.45	No Ct	ND	0.1	8.7
PMB1928	NG	C		18.22	20.07	No Ct	No Ct	22.94	No Ct	No Ct	No Ct	No Ct	ND	No Ct	ND	1.8	
PMB675	W	W		18.45	17.96	No Ct	No Ct	No Ct	17.98	No Ct	No Ct	No Ct	ND	No Ct	ND	0.5	
PMB2072	NG	W		20.52	20.15	No Ct	No Ct	No Ct	16.79	No Ct	No Ct	No Ct	ND	No Ct	ND	0.4	
PMB2913	NG	C		17.98	18.69	No Ct	No Ct	19	No Ct	No Ct	No Ct	No Ct	ND	No Ct	ND	0.7	
PMB3	NG	Y		17.85	17.71	No Ct	No Ct	No Ct	No Ct	No Ct	18.16	No Ct	ND	No Ct	ND	0.1	
PMB2344	NG	B		20.35	20.42	No Ct	18	No Ct	No Ct	No Ct	No Ct	No Ct	ND	No Ct	ND	0.1	
PMB2881	NG	C		20.14	20.03	No Ct	No Ct	16	No Ct	No Ct	No Ct	No Ct	ND	No Ct	ND	0.1	4.1
PMB1749	NG	B		18.69	18.89	No Ct	20	No Ct	No Ct	No Ct	No Ct	No Ct	ND	No Ct	ND	0.2	1.3
PMB103	NG	C		18.28	17.87	No Ct	No Ct	18	No Ct	No Ct	No Ct	34.78	ND	No Ct	ND	0.4	16.5
PMB1024	NG	B		20.22	19.93	No Ct	21	No Ct	No Ct	No Ct	No Ct	No Ct	ND	No Ct	ND	0.3	0.8
PMB1858	NG	B		18.17	18.01	No Ct	19	No Ct	No Ct	No Ct	No Ct	No Ct	ND	No Ct	ND	0.2	0.8
PMB3380	NG	W		21.33	20.98	No Ct	No Ct	No Ct	18.7	No Ct	No Ct	No Ct	ND	No Ct	ND	0.3	2.6
PMB3176	B	B		17.32	17.11	No Ct	16.17	No Ct	No Ct	No Ct	No Ct	32.49	ND	No Ct	ND	0.2	15.2
PMB1340	NG	C		18.17	18.29	No Ct	No Ct	18	No Ct	No Ct	No Ct	No Ct	ND	No Ct	ND	0.1	

Strain	Group (SASG)	GGroup rt-PCR	cnl PCR	porA Ct	ctrA Ct	NmA Ct	NmB Ct	NmC Ct	NmW Ct	NmX Ct	NmY Ct	NmE Ct*	NmE Ct	NmZ Ct*	NmZ Ct	porA -ctrA	Delta Ct
PMB2238	NG	Y		20.72	20.41	No Ct	No Ct	No Ct	No Ct	No Ct	17.78	No Ct	ND	No Ct	ND	0.3	
PMB1642	B	B		17.35	19.88	No Ct	15.64	No Ct	No Ct	No Ct	No Ct	37.11	ND	No Ct	ND	2.5	
PMB6	NG	W		20.60	20.36	No Ct	No Ct	No Ct	19.32	No Ct	No Ct	No Ct	ND	No Ct	ND	0.2	
PMB2811	NG	W		20.69	20.11	No Ct	No Ct	No Ct	21.52	No Ct	No Ct	No Ct	ND	No Ct	ND	0.6	
PMB2630	NG	B		18.15	18.18	No Ct	19	No Ct	No Ct	No Ct	No Ct	No Ct	ND	No Ct	ND	0.0	
PMB1779	NG	Y		19.75	19.84	No Ct	No Ct	No Ct	38.45	No Ct	17.76	No Ct	ND	No Ct	ND	0.1	
PMB607	B	B		16.95	17.05	No Ct	15.50	No Ct	No Ct	32.33	No Ct	No Ct	ND	No Ct	ND	0.1	15.4
PMB3563	NG	E		19.88	19.83	No Ct	36.00	No Ct	No Ct	No Ct	No Ct	20.24	17.43	No Ct	ND	0.1	
PMB2853	W	W		15.57	15.54	No Ct	No Ct	No Ct	15.65	No Ct	No Ct	No Ct	ND	No Ct	ND	0.0	
PMB628	NG	Y		21.58	21.15	No Ct	No Ct	No Ct	No Ct	No Ct	21	No Ct	ND	No Ct	ND	0.4	
PMB847	B	B		15.68	15.40	No Ct	15.64	No Ct	No Ct	No Ct	No Ct	No Ct	ND	No Ct	ND	0.3	
PMB1250	B	B		15.06	15.00	No Ct	15.21	No Ct	No Ct	No Ct	No Ct	No Ct	ND	No Ct	ND	0.1	
PMB442	Y	W/Y		23.46	23.23	No Ct	No Ct	No Ct	25.68	No Ct	28.24	No Ct	ND	No Ct	ND	0.2	4.8
PMB2661	NG	C		18.43	18.02	No Ct	No Ct	17	No Ct	No Ct	No Ct	No Ct	ND	No Ct	ND	0.4	
PMB2236	B	B		15.53	15.61	No Ct	15.97	No Ct	No Ct	No Ct	No Ct	No Ct	ND	No Ct	ND	0.1	
PMB3707	B	B		19.03	17.33	No Ct	18.28	No Ct	No Ct	No Ct	No Ct	No Ct	ND	No Ct	ND	1.7	
PMB2647	NG	B		20.05	20.01	No Ct	19.78	No Ct	No Ct	No Ct	No Ct	No Ct	ND	No Ct	ND	0.0	
PMB1933	B	B		16.91	16.46	No Ct	16.65	No Ct	No Ct	No Ct	No Ct	No Ct	ND	No Ct	ND	0.4	
PMB1491	NG	B		19.54	19.07	No Ct	19.55	No Ct	No Ct	No Ct	No Ct	No Ct	ND	No Ct	ND	0.5	
PMB3306	NG	B		21.03	21.03	No Ct	21.08	No Ct	No Ct	No Ct	No Ct	38.23	ND	No Ct	ND	0.0	
PMB3646	B	B		15.87	15.83	No Ct	15.04	No Ct	No Ct	No Ct	No Ct	No Ct	ND	No Ct	ND	0.0	
PMB2877	NG	Y		22.43	22.31	No Ct	No Ct	No Ct	No Ct	No Ct	21.03	No Ct	ND	No Ct	ND	0.1	
PMB1190	NG	Y		23.12	22.81	No Ct	No Ct	No Ct	No Ct	No Ct	22.17	No Ct	ND	No Ct	ND	0.3	
PMB472	NG	W		21.77	21.19	No Ct	No Ct	No Ct	19.83	No Ct	No Ct	38.2	ND	No Ct	ND	0.6	
PMB3083	NG	Z		22.04	22.02	No Ct	37.76	No Ct	No Ct	No Ct	No Ct	37.41	No Ct	19.54	15.81	0.0	
PMB513	B	B		15.74	15.62	No Ct	15.02	No Ct	No Ct	No Ct	No Ct	No Ct	ND	No Ct	ND	0.1	
PMB229	Y	Y		23.02	22.62	No Ct	No Ct	No Ct	No Ct	No Ct	24.53	No Ct	ND	No Ct	ND	0.4	
PMB3437	W	W		16.09	15.84	No Ct	No Ct	No Ct	15.7	No Ct	No Ct	No Ct	ND	No Ct	ND	0.2	
PMB2167	B	B		16.44	16.56	No Ct	16.33	No Ct	No Ct	No Ct	No Ct	No Ct	ND	No Ct	ND	0.1	

Strain	Group (SASG)	GGroup rt-PCR	cnl PCR	porA Ct	ctrA Ct	NmA Ct	NmB Ct	NmC Ct	NmW Ct	NmX Ct	NmY Ct	NmE Ct*	NmE Ct	NmZ Ct*	NmZ Ct	porA -ctrA	Delta Ct
PMB3145	NG	X		23.92	23.72	No Ct	No Ct	No Ct	No Ct	25.19	No Ct	No Ct	ND	No Ct	ND	0.2	
PMB2593	NG	B		22.74	22.48	No Ct	19.72	No Ct	No Ct	38.51	No Ct	38.35	ND	No Ct	ND	0.3	
PMB1394	B	B		14.56	14.82	No Ct	15.26	No Ct	No Ct	No Ct	No Ct	No Ct	ND	No Ct	ND	0.3	
PMB2977	B	B		16.21	16.06	No Ct	16.23	No Ct	No Ct	No Ct	No Ct	No Ct	ND	No Ct	ND	0.2	
PMB2025	NG	B		23.76	23.27	No Ct	21.98	No Ct	No Ct	No Ct	No Ct	No Ct	ND	No Ct	ND	0.5	
PMB1790	B	B		15.52	15.56	No Ct	15.54	No Ct	No Ct	No Ct	No Ct	No Ct	ND	No Ct	ND	0.0	
PMB2138	NG	B		23.21	23.03	No Ct	20.92	No Ct	No Ct	No Ct	No Ct	No Ct	ND	No Ct	ND	0.2	
PMB3659	W	W		16.07	15.84	No Ct	No Ct	No Ct	16.13	No Ct	No Ct	No Ct	ND	No Ct	ND	0.2	
PMB542	NG	B		20.45	20.03	No Ct	21.27	No Ct	No Ct	No Ct	No Ct	No Ct	ND	No Ct	ND	0.4	
PMB3405	NG	W		18.84	18.47	No Ct	No Ct	No Ct	19.06	No Ct	No Ct	No Ct	ND	No Ct	ND	0.4	
PMB2016	NG	W		16.77	17.62	No Ct	No Ct	No Ct	18.73	No Ct	No Ct	37.43	ND	No Ct	ND	0.8	
PMB1806	NG	W		19.12	19.04	No Ct	No Ct	No Ct	20.05	39.78	No Ct	No Ct	ND	No Ct	ND	0.1	
PMB318	X	X		15.10	15.07	No Ct	36.22	No Ct	No Ct	15.96	No Ct	No Ct	ND	No Ct	ND	0.0	
PMB1736	X	Y		17.28	16.14	No Ct	36.24	No Ct	No Ct	No Ct	17.00	No Ct	ND	No Ct	ND	1.1	
PMB3113	NG	C		18.67	18.40	No Ct	39.78	18.31	No Ct	No Ct	No Ct	No Ct	ND	No Ct	ND	0.3	
PMB743	NG	Y		17.28	17.43	No Ct	No Ct	37.62	No Ct	No Ct	18.51	No Ct	ND	No Ct	ND	0.2	
PMB1442	NG	B		19.51	19.46	No Ct	19.77	No Ct	No Ct	No Ct	No Ct	No Ct	ND	No Ct	ND	0.1	
PMB2869	B	B		16.81	16.95	No Ct	17.23	32.30	No Ct	No Ct	35.17	38.94	ND	No Ct	ND	0.1	15.5
PMB2829	NG	B		19.01	18.84	No Ct	18.39	No Ct	No Ct	No Ct	No Ct	No Ct	ND	No Ct	ND	0.2	
PMB3235	B	B		15.93	15.88	No Ct	16.19	No Ct	No Ct	No Ct	No Ct	No Ct	ND	No Ct	ND	0.1	
PMB2293	Y	Y		22.69	22.42	No Ct	38.33	No Ct	No Ct	No Ct	24.74	No Ct	ND	No Ct	ND	0.3	
PMB553	NG	E		19.86	19.49	No Ct	No Ct	34	No Ct	No Ct	No Ct	19.56	18.35	No Ct	ND	0.4	14.1
PMB1570	B	B		16.20	15.87	No Ct	15.86	No Ct	No Ct	No Ct	No Ct	No Ct	ND	No Ct	ND	0.3	
PMB456	E	E		22.26	22.13	No Ct	No Ct	No Ct	No Ct	No Ct	No Ct	17.21	13.61	No Ct	ND	0.1	
PMB3255	NG	E		17.63	19.74	No Ct	35.00	No Ct	No Ct	No Ct	29	16.50	15.62	No Ct	ND	2.1	17.4
PMB560	NG	C		20.50	20.17	No Ct	30.00	19	No Ct	No Ct	No Ct	No Ct	ND	No Ct	ND	0.3	9.5
PMB2442	NG	B		19.11	19.03	No Ct	19.44	No Ct	No Ct	No Ct	No Ct	29.41	No Ct	37.6	ND	0.1	10.3
PMB1983	B	B		15.56	15.32	No Ct	15.02	No Ct	No Ct	No Ct	No Ct	38.05	ND	No Ct	ND	0.2	
PMB173	NG	E		22.78	22.66	No Ct	No Ct	No Ct	No Ct	No Ct	No Ct	20.26	18.09	No Ct	ND	0.1	

Strain	Group (SASG)	GGroup rt-PCR	cnl PCR	porA Ct	ctrA Ct	NmA Ct	NmB Ct	NmC Ct	NmW Ct	NmX Ct	NmY Ct	NmE Ct*	NmE Ct	NmZ Ct*	NmZ Ct	porA -ctrA	Delta Ct
PMB2359	NG	B		20.29	20.31	No Ct	20.34	No Ct	No Ct	No Ct	No Ct	No Ct	ND	No Ct	ND	0.0	
PMB819	B	B		19.79	19.48	No Ct	19.26	No Ct	No Ct	No Ct	No Ct	No Ct	ND	No Ct	ND	0.3	
PMB3123	NG	C		25.30	25.15	No Ct	No Ct	18	No Ct	No Ct	No Ct	29.54	37	No Ct	ND	0.2	11.7
PMB3392	B	B		17.11	16.76	No Ct	15.65	No Ct	No Ct	No Ct	No Ct	No Ct	ND	No Ct	ND	0.3	
PMB2859	Y	Y		22.81	22.29	No Ct	No Ct	No Ct	No Ct	No Ct	24.5	No Ct	ND	No Ct	ND	0.5	
PMB1501	NG	Y		29.12	29.17	No Ct	No Ct	No Ct	No Ct	No Ct	18.79	22.93	37	No Ct	ND	0.0	
PMB2111	B	B		15.58	15.66	No Ct	15.95	26.12	No Ct	No Ct	No Ct	No Ct	ND	No Ct	ND	0.1	10.5
PMB2999	C	C		14.28	14.89	No Ct	33.57	14.85	No Ct	No Ct	No Ct	No Ct	ND	No Ct	ND	0.6	19.3
PMB843	W	W		15.63	19.45	No Ct	No Ct	33.99	20.03	No Ct	No Ct	38.17	ND	No Ct	ND	3.8	18.4
PMB557	W	W		16.24	15.71	No Ct	No Ct	No Ct	15.92	No Ct	No Ct	No Ct	ND	No Ct	ND	0.5	
PMB1799	B	B		15.98	16.04	No Ct	15.96	No Ct	35.1	No Ct	No Ct	No Ct	ND	No Ct	ND	0.1	
PMB158	W	W		16.64	16.22	No Ct	No Ct	No Ct	16.16	No Ct	No Ct	No Ct	ND	No Ct	ND	0.4	
PMB2224	W	W		16.31	16.08	No Ct	No Ct	No Ct	16.26	No Ct	No Ct	No Ct	ND	No Ct	ND	0.2	
PMB3273	NG	B		17.03	17.02	No Ct	16.42	No Ct	No Ct	No Ct	No Ct	No Ct	ND	No Ct	ND	0.0	
PMB609	B	B		15.33	15.34	No Ct	15.64	No Ct	31.99	No Ct	No Ct	No Ct	ND	No Ct	ND	0.0	16.7
PMB809	W	W		15.64	15.58	No Ct	37.05	No Ct	15.76	No Ct	No Ct	No Ct	ND	No Ct	ND	0.1	
PMB2474	W	W		15.24	15.34	No Ct	No Ct	35.15	15.55	No Ct	No Ct	No Ct	ND	No Ct	ND	0.1	
PMB2328	NG	B		19.47	No Ct	No Ct	19.74	No Ct	No Ct	No Ct	No Ct	No Ct	ND	No Ct	ND	NA	
PMB2787	C	C		16.01	16.20	No Ct	28.8	16.45	33.44	No Ct	No Ct	No Ct	ND	No Ct	ND	0.2	17.4
PMB2849	B	B		16.22	15.99	No Ct	16.27	36.28	No Ct	No Ct	No Ct	No Ct	ND	No Ct	ND	0.2	
PMB3187	NG	B		20.46	20.24	No Ct	20.85	No Ct	No Ct	No Ct	No Ct	No Ct	ND	No Ct	ND	0.2	
PMB2617	NG	B		15.82	16.19	No Ct	17.32	24.8	No Ct	No Ct	No Ct	No Ct	ND	No Ct	ND	0.4	9.0
PMB3500	NG	Y		20.38	20.24	No Ct	31.20	37.66	No Ct	No Ct	18.6	No Ct	ND	No Ct	ND	0.1	10.8
PMB1763	NG	C		20.87	20.67	No Ct	No Ct	18.59	No Ct	No Ct	No Ct	No Ct	ND	No Ct	ND	0.2	
PMB463	NG	Y		15.03	15.13	No Ct	32.29	24.84	27.79	No Ct	16.47	38.58	ND	No Ct	ND	0.1	17.3
PMB842	NG	W		20.96	20.39	No Ct	36.92	No Ct	18.01	No Ct	No Ct	No Ct	ND	No Ct	ND	0.6	
PMB3214	NG	Y		20.36	20.52	No Ct	38.26	No Ct	No Ct	No Ct	18.57	No Ct	ND	No Ct	ND	0.2	
PMB397	NG	Z		22.58	22.52	No Ct	36.78	35.34	No Ct	No Ct	No Ct	34.55	No Ct	18.81	14.42	0.1	12.0
PMB608	NG	W		20.21	19.73	No Ct	33.23	38.18	18.02	No Ct	No Ct	No Ct	ND	No Ct	ND	0.5	13.0

Strain	Group (SASG)	GGroup rt-PCR	cnl PCR	porA Ct	ctrA Ct	NmA Ct	NmB Ct	NmC Ct	NmW Ct	NmX Ct	NmY Ct	NmE Ct*	NmE Ct	NmZ Ct*	NmZ Ct	porA -ctrA	Delta Ct
PMB1900	NG	B		15.16	15.32	No Ct	17.94	29.46	No Ct	No Ct	No Ct	No Ct	ND	No Ct	ND	0.2	14.3
PMB1016	NG	E		21.23	23.43	No Ct	No Ct	32.91	38.96	No Ct	No Ct	17.54	16.42	No Ct	ND	2.2	11.7
PMB3323	NG	Y		20.73	20.33	No Ct	No Ct	No Ct	No Ct	No Ct	18.34	No Ct	ND	No Ct	ND	0.4	
PMB667	NG	Z		21.61	21.47	No Ct	No Ct	No Ct	No Ct	No Ct	35.49	35.34	No Ct	18.47	15.58	0.1	
PMB1894	NG	E		24.09	23.84	No Ct	No Ct	No Ct	No Ct	No Ct	No Ct	22.15	21.5	No Ct	ND	0.2	
PMB596	NG	C		21.89	21.72	No Ct	39.87	19.97	34.17	No Ct	No Ct	No Ct	ND	No Ct	ND	0.2	12.3
PMB1266	NG	W		20.79	20.25	No Ct	No Ct	No Ct	18.60	No Ct	No Ct	No Ct	ND	No Ct	ND	0.5	
PMB834	NG	B		20.74	20.53	No Ct	18.47	No Ct	26.28	No Ct	No Ct	No Ct	ND	No Ct	ND	0.2	5.5
PMB1713	NG	E		22.06	24.40	No Ct	No Ct	32.77	No Ct	No Ct	No Ct	18.09	19.25	No Ct	ND	2.3	10.7
PMB2429	NG	Z		21.95	21.82	No Ct	28.87	No Ct	No Ct	No Ct	No Ct	No Ct	No Ct	19.25	19.02	0.1	6.9
PMB2607	NG	W		20.64	20.29	No Ct	38.01	No Ct	19.37	No Ct	No Ct	No Ct	ND	No Ct	ND	0.4	
PMB2023	NG	C		22.28	21.82	No Ct	34.82	20.5	No Ct	No Ct	No Ct	No Ct	ND	No Ct	ND	0.5	12.5
PMB273	NG	W		12.07	9.73	No Ct	24.45	23.71	11.19	No Ct	No Ct	No Ct	ND	No Ct	ND	2.3	12.5
PMB1245	NG	Y/C		16.49	16.65	No Ct	39.43	24.78	No Ct	No Ct	23.47	No Ct	ND	No Ct	ND	0.2	1.3
PMB1652	NG	B		21.31	21.33	No Ct	19.84	No Ct	No Ct	No Ct	No Ct	No Ct	ND	No Ct	ND	0.0	
PMB3521	NG	B		20.37	20.17	No Ct	18.66	No Ct	No Ct	No Ct	No Ct	32.62	ND	No Ct	ND	0.2	12.2
PMB1972	NG	Y		21.78	21.38	No Ct	36.29	No Ct	No Ct	No Ct	19.96	No Ct	ND	No Ct	ND	0.4	
PMB981	NG	E		20.86	20.53	No Ct	No Ct	No Ct	No Ct	No Ct	No Ct	16.79	15.89	No Ct	ND	0.3	
PMB2216	NG	E		22.32	24.64	No Ct	No Ct	No Ct	No Ct	No Ct	No Ct	20.13	16.06	No Ct	ND	2.3	
PMB1343	NG	B		21.04	20.45	No Ct	18.94	No Ct	No Ct	No Ct	No Ct	No Ct	ND	No Ct	ND	0.6	
PMB739	NG	W		20.26	19.66	No Ct	No Ct	No Ct	17.09	No Ct	No Ct	No Ct	ND	No Ct	ND	0.6	
PMB1363	NG	E		21.66	21.60	No Ct	34.2	No Ct	No Ct	No Ct	No Ct	18.82	15.43	No Ct	ND	0.1	12.5
PMB101	NG	Y		21.77	21.74	No Ct	No Ct	No Ct	No Ct	No Ct	20.33	No Ct	ND	No Ct	ND	0.0	
PMB1359	NG	E		23.01	25.01	No Ct	No Ct	No Ct	No Ct	No Ct	No Ct	21.31	19.57	No Ct	ND	2.0	
PMB3150	NG	C		21.83	21.53	No Ct	35.9	19.45	No Ct	No Ct	No Ct	36.48	ND	No Ct	ND	0.3	
PMB275	NG	Y		23.07	23.04	No Ct	No Ct	No Ct	39.72	No Ct	24.25	No Ct	ND	No Ct	ND	0.0	
PMB1030	NG	E		27.57	29.96	No Ct	No Ct	No Ct	No Ct	No Ct	No Ct	29.03	28.31	No Ct	ND	2.4	
PMB3619	NG	W		22.62	22.09	No Ct	No Ct	No Ct	23.7	No Ct	No Ct	38.38	ND	No Ct	ND	0.5	
PMB1598	NG	C		15.52	15.61	No Ct	37.11	10.50	No Ct	No Ct	No Ct	No Ct	ND	No Ct	ND	0.1	

Strain	Group (SASG)	GGroup rt-PCR	cnl PCR	porA Ct	ctrA Ct	NmA Ct	NmB Ct	NmC Ct	NmW Ct	NmX Ct	NmY Ct	NmE Ct*	NmE Ct	NmZ Ct*	NmZ Ct	porA -ctrA	Delta Ct
PMB1871	NG	C		22.88	22.73	No Ct	No Ct	23.47	No Ct	No Ct	No Ct	No Ct	ND	No Ct	ND	0.2	
PMB417	NG	E		21.88	23.22	No Ct	No Ct	No Ct	33.03	No Ct	No Ct	17.54	15.43	No Ct	ND	1.3	11.1
PMB879	NG	E		13.97	15.24	No Ct	No Ct	No Ct	No Ct	No Ct	No Ct	18.42	18.07	No Ct	ND	1.3	

NG: nongroupable using SASG assay
NGG: nongenogroupable using rt-PCR assays
cnl: positive PCR product (<400bp) from *tex* to *galE*
NR: negative *cnl* PCR and negative rt-PCR
ND: not done
*: Results with MenE and MenZ specific primer-probes located at 5'end of *ctrA*
Delta Ct: difference in Ct values between *porA* and genogrouping assay with Ct of 30–35.
