# Supplementary material for: Optimization of Molecular Approaches to Genogroup Neisseria meningitidis Carriage Isolates and Implications for Monitoring the Impact of New Serogroup B Vaccines
Source: PLoS One. 2015 Jul 6;10(7):e0132140. doi: 10.1371/journal.pone.0132140 (PMC4493136; doi:10.1371/journal.pone.0132140)
Supplement: S3 Table — (PDF) [file pone.0132140.s003.pdf]

S3 Table. Specificity of meningococcal genogrouping primer-probe combinations.

**Strain**	**Serogroup**	***porA***	***ctrA***	**NmA**	**NmB**	**NmC**	**NmE**	**NmW**	**NmX**	**NmY**	**NmZ**
A1	A	19.40	18.68	19.50	No Ct	No Ct	No Ct	No Ct	No Ct	No Ct	No Ct
F8238	A	19.25	18.21	19.17	No Ct	No Ct	No Ct*	No Ct	No Ct	No Ct	No Ct
MC58	B	18.90	17.69	No Ct	18.27	No Ct	No Ct	No Ct	No Ct	No Ct	No Ct
CDC1573	B	16.12	14.73	No Ct	18.13	No Ct	No Ct	No Ct	No Ct	No Ct	No Ct*
C11	C	16.49	15.56	No Ct	No Ct	16.54	No Ct	No Ct	No Ct	No Ct	No Ct
ATCC 35558	E	16.17	15.58	No Ct	No Ct	No Ct	16.01	No Ct	No Ct	No Ct	No Ct
ATCC 35559	W	16.85	15.70	No Ct	No Ct	No Ct	No Ct	14.53	No Ct	No Ct	No Ct
ATCC 35559[a]	W	17.72	16.77	No Ct	No Ct	No Ct	No Ct	16.02	No Ct	No Ct	No Ct
ATCC 35560	X	18.32	17.14	No Ct	No Ct	No Ct	No Ct	No Ct	16.41	No Ct	No Ct
ATCC 35561	Y	17.24	15.89	No Ct	No Ct	No Ct	No Ct	No Ct	No Ct	16.32	No Ct
M6130	Y	19.91	18.70	No Ct	No Ct	No Ct	No Ct	No Ct*	No Ct	19.58	No Ct
M6043	Y	16.80	15.93	No Ct	No Ct	No Ct*	No Ct	No Ct	No Ct	16.56	No Ct
ATCC 35562	Z	16.39	16.04	No Ct	No Ct	No Ct	34.50	No Ct	No Ct	No Ct	16.84

Ct values represent the average of two replicates
*One of the replicates in these cells had a Ct >38
[a]Strain ATCC 35559 grown in different media
